# Supplementary material for: Efficacy and safety of PM-AR-T versus edwards MC3 rings in tricuspid regurgitation: A non-inferiority, randomized controlled trial
Source: PLoS One. 2025 Dec 12;20(12):e0333891. doi: 10.1371/journal.pone.0333891 (PMC12700415; doi:10.1371/journal.pone.0333891)
Supplement: S1 Section — (DOCX) [file pone.0333891.s003.docx]

S1 Section. PM-AR-T introduction

1. Device characteristics

The PM-AR-T annuloplasty ring features a tubular base frame constructed from nickel-titanium (NiTi) alloy, conforming to a three-dimensional elliptical contour that replicates the physiological morphology of the tricuspid annulus. Mounted on a dedicated annuloplasty holder for facilitated implantation, this device is clinically indicated for corrective remodeling of pathological valve annuli and maintenance of optimal leaflet coaptation area (Supplementary Figure 1).

1. Structural composition, operating principle, and mechanism of action

The PM-AR-T annuloplasty ring is constructed from a nickel-titanium (NiTi) alloy tube shaped into a closed loop, with its connection point secured by nickel-titanium (NiTi) alloy wire. The middle layer features a silicone material that includes a suture flange, while the space between this silicone layer and the base is filled with a long-term implantable silicone substance. The outer layer is made of polyester fabric, which offers excellent biocompatibility.

This PM-AR-T annuloplasty ring is specifically designed for tricuspid valve repair and has an oval shape that conforms to a normal tricuspid valve orifice. For ease of implantation, the ring is attached to an annuloplasty ring holder, both of which come in sterile packaging for single-use.

The PM-AR-T annuloplasty ring is intended to be used with the annuloplasty ring holder handle and the ring sizer. These components are supplied in separate, non-sterile packaging and need to be cleaned and sterilized using high-temperature steam before use. They can be reused multiple times.
