# Supplementary material for: Efficacy and safety of PM-AR-T versus edwards MC3 rings in tricuspid regurgitation: A non-inferiority, randomized controlled trial
Source: PLoS One. 2025 Dec 12;20(12):e0333891. doi: 10.1371/journal.pone.0333891 (PMC12700415; doi:10.1371/journal.pone.0333891)
Supplement: S1 Table — (DOCX) [file pone.0333891.s004.docx]

S1 Table. Comparative specifications of multi-model devices

| Specifications and models | Edwards MC3 | | | PM-AR-T | | |
| --- | --- | --- | --- | --- | --- | --- |
|  | Maximum diameter, mm | Height, mm | Area, mm | Maximum diameter, mm | Height, mm | Area, mm |
| T26 | 28.43 | 4.00 | 419.44 | 33 | 7 | 318 |
| T28 | 29.86 | 4.37 | 475.68 | 35 | 7 | 386 |
| T30 | 32.47 | 4.86 | 554.00 | 37 | 7 | 460 |
| T32 | 34.04 | 4.66 | 636.21 | 39 | 7 | 539 |
| T34 | 34.99 | 5.92 | 722.15 | 41 | 7 | 626 |
| T36 | 37.02 | 6.02 | 802.15 | 43 | 7 | 720 |
