# Supplementary figures and images for: Efficacy and safety of PM-AR-T versus edwards MC3 rings in tricuspid regurgitation: A non-inferiority, randomized controlled trial
Source: PLoS One. 2025 Dec 12;20(12):e0333891. doi: 10.1371/journal.pone.0333891 (PMC12700415; doi:10.1371/journal.pone.0333891)

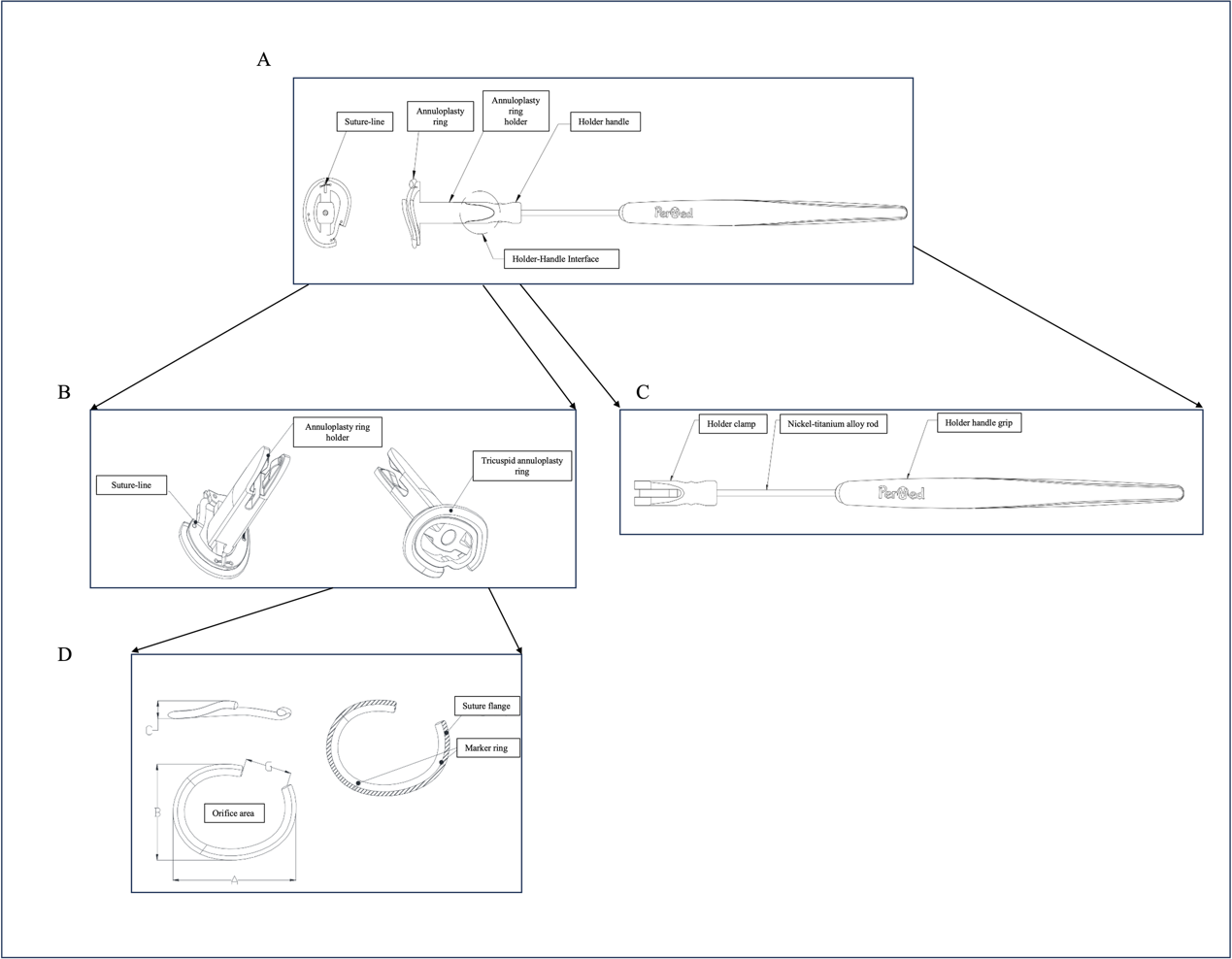

Supplement: S1 Fig — A. Device assembly schematic. B. Composite schematic: annuloplasty ring with holder. C. Holder handle assembly schematic. D. Annuloplasty ring. (TIF) [file pone.0333891.s005.tif]

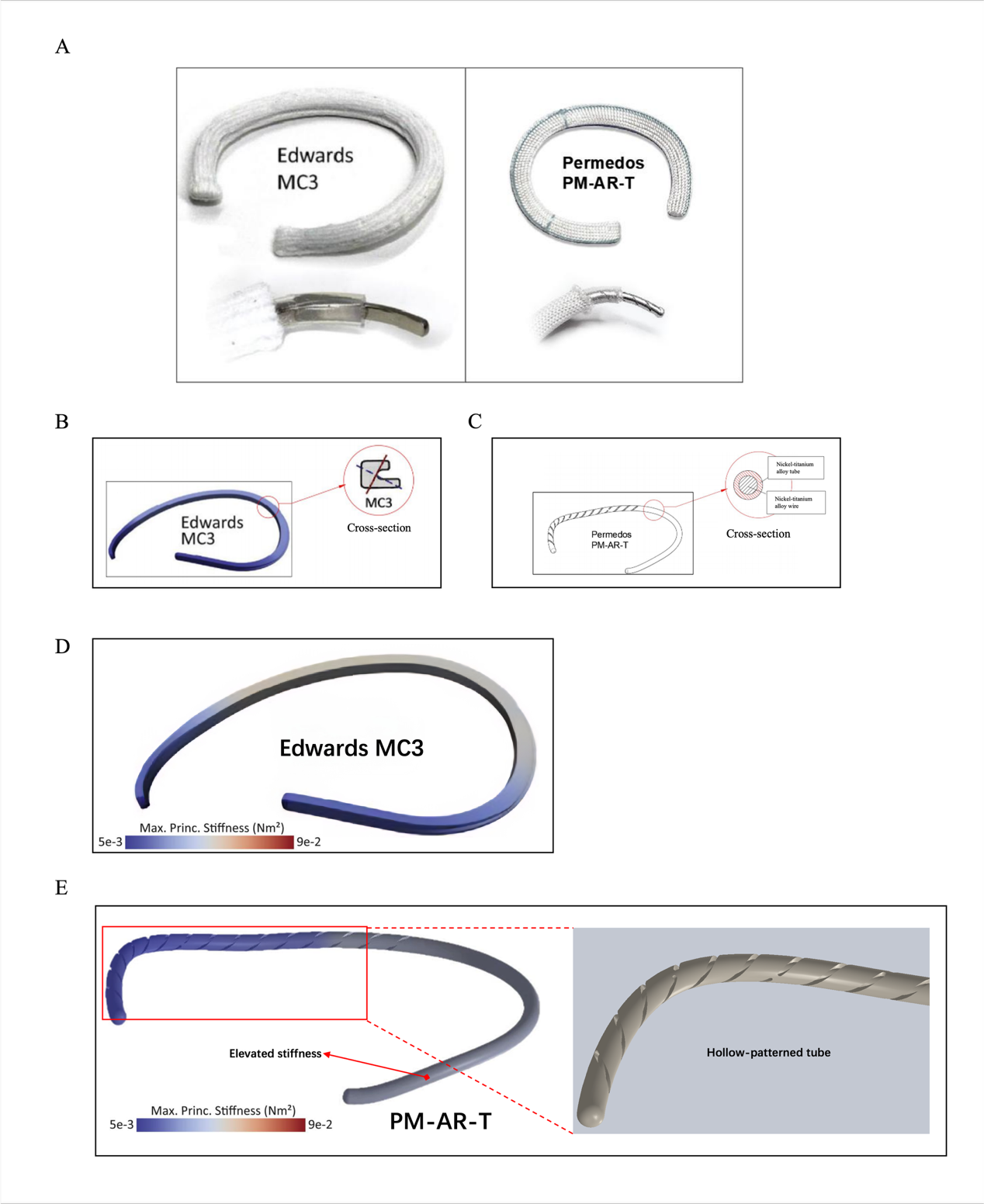

Supplement: S2 Fig — A. Overview of Edwards MC3 and PM-AR-T tricuspid annuloplasty rings. B. Cross-section of the Edwards MC3 tricuspid annuloplasty ring. C. Cross-section of the PM-AR-T tricuspid annuloplasty ring. D. Stiffness distribution map of Edwards MC3. E. Stiffness distribution map of the PM-AR-T and an enlarged view of its hollow-patterned tube. (TIF) [file pone.0333891.s006.tif]
