# Supplementary material for: Efficacy and safety of PM-AR-T versus edwards MC3 rings in tricuspid regurgitation: A non-inferiority, randomized controlled trial
Source: PLoS One. 2025 Dec 12;20(12):e0333891. doi: 10.1371/journal.pone.0333891 (PMC12700415; doi:10.1371/journal.pone.0333891)
Supplement: S2 File — (PDF) [file pone.0333891.s008.pdf]

Clinical trial protocol for medical devices

Protocol Number: TAR-CT (CN)

Trial name: A prospective, multicenter, randomized controlled clinical trial to evaluate the safety and efficacy of Annuloplasty Ring in the treatment of tricuspid regurgitation

Investigational Device: Annuloplasty Ring (Beijing Permed Biomedical Engineering Co..Ltd)

Model specification: sizes 26、28、30、32、34、36

Control Device: Edwards MC3 Ring（Edwards Lifesciences (Shanghai) Medical Products Co., Ltd.）

Model specification: sizes T26－36

Management category of medical devices for testing:

Is it a class III medical device that needs clinical trial approval? ☐Yes. ☑No

Are there similar products in China? ☑Yes.☐No.

Version Number and Date of the Protocol: V1.2/2022-01-12

Clinical trial institution: The Affiliated Hospital of Nanjing University Medical School

Coordinating researcher: Wang Dongjin MD

Statistics director: Zheng Qingshan MD

Data management and statistical analysis unit: Shanghai Bojia Pharmaceutical Technology Co., LTD

Sponsor: Beijing Permed Biomedical Engineering Co..Ltd.

Specification

1. For multicenter clinical trials, only the leading organization is filled in on the cover, and other organizations are listed in the protocol content.

2. For multicenter clinical trials, the investigator on the cover is the coordinating investigator.

Contents

Protocol Synopsis....5
1 .Sponsor Information....12
2 . List of all clinical trial institutions and investigators for multicenter clinical trial:12
3. Purpose and content of clinical trial....13
3.1Object....13
3.2Content....13
4. Background data of clinical trials....15
4.1 Study rationale....15
4.2 Study background....16
5.Product Features, Structural Composition, Working Principle, Mechanism of Action, and Testing Scope....23
5.1 Product Features....24
5.2 Structural Composition, Working Principle, and Mechanism of Action....24
5.3 Testing Scope Applicable to patients with tricuspid regurgitation who are eligible for Annuloplasty Ring implantation.....25
6. Indications, Contraindications, Precautions....25
6.1 Indications The Annuloplasty Ring is indicated for:Correction of annular dilation.Enhancement of leaflet coaptation and annular stabilization.Prevention of further annular expansion.....25
6.2 Contraindications....25
6.3 Precautions....25
6.4 Storage and Transportation....26
6.5 Complications....26
7. Overall Trial Design....28
7.1 Trial Design....28
7.2 Trial Procedures....44
7.3 Monitoring Plan....47
8. Statistical Considerations....48
8.1 Statistical Design, Methods, and Analytical Procedures....48
8.2 Calculation of sample size....51
8.3 Significance level and confidence level of clinical trials....51
8.4 Expected fall-off rate....52
8.5 Criteria for qualified/unqualified clinical trial results....52
8.6 Criteria and reasons for terminating the trial on statistical grounds....52
8.7 Statistical methods for all data, including processing of missing, unused or erroneous data (including dropout and withdrawal) and unreasonable data....52
8.8 Report deviations from the planned statistical procedures....52
8.9 Selection criteria and reasons for subjects included in the analysis....53
8.10 Exclusion of special information and its rationale in the verification of assumptions (if applicable)....53
9. Data management....53
9.1 EDC data management....53
9.2 External data transfer....54
9.3 Medical coding....54
10. Feasibility analysis....55
10.1 Analysis of the likelihood of success....55
10.2 Analysis of the possibility of failure....55

11. Quality control of clinical trials 56
    11.1 Training for clinical trials........ 56
    11.2 Monitoring of clinical trials 56
    11.3 Audit of clinical trials 56
    11.4 Preservation of original data 56
12. Ethical issues and informed consent in clinical trials 56
    12.1 Ethical considerations 56
    12.2 Approval of the test plan 57
    12.3 Process of informed consent and text of informed consent form 57
13. Regulations on the reporting of adverse events and device defects 58
    13.1 Adverse events........ 58
    13.2 Serious adverse events and device defects........ 59
    13.3 Reporting procedures and contact information 59
14. Deviation from and amendment of the clinical trial protocol 60
15. Direct access to source data and files 60
16. Finance and insurance 60
17. Contents that should be covered in clinical trial reports 60
18. Confidentiality principle........ 61
19. Agreement on publication of test results 61
20.Duties of the parties........ 62
    20.1 Responsibilities of the sponsor 62
    20.2 Responsibilities of clinical trial institutions and investigators 64
References........ 66

Protocol Synopsis

**Name of the trial**	A prospective, multicenter, randomized, controlled clinical trial evaluating the safety and efficacy of heart valve prosthetic rings for the treatment of tricuspid regurgitation
**Test equipment**	Annuloplasty Ring
**Experiment al stage**	Clinical validation of Class III medical devices
**Research design**	Prospective, multicenter, randomized controlled, non-inferior
**Test equipment**	Investigational Device: Annuloplasty Ring (Beijing Permed Biomedical Engineering Co..Ltd)<br>Control Device: Edwards MC3 Ring（Edwards Lifesciences (Shanghai) Medical Products Co., Ltd.）
**Sample capacity**	164 cases
**Study population**	Patients with tricuspid valve insufficiency.
**Test objective**	To evaluate the safety and efficacy of cardiac annuloplasty ring developed by Permed Biomedical Engineering Co., Ltd for the treatment of tricuspid insufficiency.
**Primary endpoint**	The success rate of valve annuloplasty repair at 6 months postoperatively, defined as no severe regurgitation and an increase in diameter ≤15% (based on a comprehensive assessment of regurgitation severity and diameter increase).

**Secondary endpoint**	1 At discharge or 30 days postoperatively (whichever comes first), 3 months, 6 months, and 12 months:<br><br>1.1 Degree of valve regurgitation assessed by echocardiography (evaluated according to the tricuspid regurgitation assessment index table).<br><br>1.2 Changes in echocardiographic parameters (right atrial and ventricular size, diameter, width of the narrowest regurgitation jet, and revers.<br><br>1.3 NYHA functional class.<br><br>1.4 Freedom from reoperation due to tricuspid valve insufficiency.<br><br>2 Evaluation of the trial device's performance, including needle insertion smoothness and user-friendliness of the ring holder.<br><br>3 Technical success rate of the surgery (before leaving the operating room), defined as no intraoperative death, successful implantation of the annuloplasty ring, successful removal of the ring holder, and no emergency surgery or secondary intervention related to the trial device.<br><br>4 At discharge or 30 days postoperatively (whichever comes first), 3 months, 6 months, and 12 months:<br><br>4.1 Incidence of serious adverse events, including cardiovascular death, major bleeding, severe damage to cardiac or other body structures, infective endocarditis, and thromboembolic events related to the trial device.<br><br>4.2 All-cause mortality.<br><br>4.3 Cardiovascular death related to the trial device.<br><br>4.4 Major bleeding related to the trial device.<br><br>4.5 Severe damage to cardiac or other body structures related to the trial device.<br><br>4.6 Infective endocarditis related to the trial device.<br><br>4.7 Thromboembolic events related to the trial device.<br><br>5 Device defects.<br><br>6 Other adverse events.

Trial Design:

This trial is designed as a prospective, multicenter, randomized controlled study to evaluate the safety and efficacy of the tricuspid annuloplasty ring developed by Beijing Permed Biomedical Engineering Co..Ltd.. for the treatment of tricuspid regurgitation. According to the inclusion and exclusion criteria of this trial, at least 164 subjects will be enrolled and randomly assigned to either the experimental group or the control group. The experimental group will use the tricuspid annuloplasty ring developed by Beijing Permed Biomedical Engineering Co..Ltd.., while the control group will use the tricuspid annuloplasty ring produced by Edwards Lifesciences (Shanghai) Medical Products Co., Ltd. for tricuspid valve repair surgery. Follow-up imaging and/or clinical evaluations will be conducted intraoperatively, before discharge or within 30 ± 7 days postoperatively, at 3 ± 15 months postoperatively, at 6±30 months postoperatively, and at 12±30 months postoperatively.

The primary endpoint is the success rate of valve repair at 6 months postoperatively, defined as no severe regurgitation and an increase in annular diameter of ≤15% (based on a comprehensive assessment of regurgitation severity and annular diameter increase).

Secondary efficacy endpoints include the degree of valve regurgitation assessed by echocardiography before discharge or at 30 days postoperatively (whichever comes first), at 3 months, 6 months, and 12 months postoperatively; changes in echocardiographic parameters (right atrial and ventricular size, annular diameter, width of the narrowest regurgitant jet, and reverse flow area); NYHA functional class; freedom from reoperation due to tricuspid regurgitation; and evaluation of the device's performance, including needle passage smoothness and user-friendliness of the ring holder. The technical success rate of the surgery (before leaving the operating room) is defined as no intraoperative death, successful implantation of the annuloplasty ring, successful removal of the ring holder, and no emergency surgery or secondary intervention related to the investigational device.

Safety endpoints include the incidence of serious adverse events (including cardiovascular-related death, major bleeding, severe damage to cardiac or other body structures, infective endocarditis, and thromboembolic events related to the investigational device) before discharge or at 30 days postoperatively (whichever comes first), at 3 months, 6 months, and 12 months postoperatively; all-cause mortality; cardiovascular-related death related to the investigational device; major bleeding related to the investigational device; severe damage to cardiac or other body structures related to the

investigational device; infective endocarditis related to the investigational device; thromboembolic events related to the investigational device; device defects; and other adverse events.

The follow-up content included clinical symptoms and signs, laboratory examination, electrocardiogram, color Doppler ultrasound, recording adverse events, etc., to verify the safety and effectiveness of the heart valve forming ring used in the trial.

It is planned to complete the summary report after the 6-month postoperative follow-up and submit it to the National Medical Products Administration for product registration. After the 12-month postoperative follow-up, a one-year follow-up report will be issued, and long-term safety evaluations will be conducted at 2±30 days, 3±30 days, 4±30 days, and 5±30 days postoperatively based on this report.

**Inclusion Criteria**	To be eligible for the trial, a patient must meet all of the following criteria:<br>1 Age: Between 18 and 75 years old.<br>2 Diagnosis: Diagnosed with tricuspid valve disease (e.g., degenerative or functional valve pathology) requiring surgical repair.<br>3 Heart Function: Preoperative New York Heart Association (NYHA) functional class III or lower.<br>4 Consent: Willing and able to provide written informed consent, comply with follow-up visits, and complete required postoperative evaluations.
**Exclusion Criteria**	A patient will not be eligible if they meet any of the following conditions:<br>1 Contraindications for Annuloplasty Ring implantation or Previous tricuspid valve surgery.<br>2 Severe Heart Conditions:NYHA class IV heart failure,Right heart failure.<br>3 Researchers determine patients with increased surgical risks due to thrombus in the right atrium or right ventricle.<br>4 History of heart transplant.<br>5 Emergency surgery requirement.<br>6 Life expectancy less than 1 year.<br>7 Recent cardiac surgery, interventional procedure, or stroke within the past 3 months.<br>8 Kidney failure requiring dialysis.<br>9 Severe respiratory disease.<br>10 Active infective endocarditis.<br>11 Active systemic infection, sepsis, or unexplained fever.

	12 Active gastrointestinal bleeding , peptic ulcers.or hypercoagulability in the blood.<br><br>13 Contraindications to anticoagulants/antiplatelet therapy.<br><br>14 Allergies: Known hypersensitivity to nitinol, silicone, or polyester materials.<br><br>15 Pregnancy: Currently pregnant or breastfeeding.<br><br>16 Other Trials: Participation in another drug or device study.<br><br>17 Miscellaneous: Any condition deemed unsuitable for enrollment by the investigator.
**Trial Procedures**	**1 Screening (within 30 days preoperatively)**<br><br>1.1 Patients must sign the informed consent form before screening.<br><br>1.2 Collect demographic data (gender, date of birth, weight, and height).<br><br>1.3 NYHA functional class, vital signs (blood pressure, pulse).<br><br>1.4 Laboratory tests: Complete blood count, blood biochemistry, coagulation function, and pregnancy test if necessary.<br><br>1.5 Electrocardiogram (ECG), echocardiography (TTE or TEE).<br><br>1.6 Record antiplatelet or anticoagulant medications.<br><br>1.7 Randomization using a central randomization system.<br><br>**2 Surgical Treatment (Day 0, Surgery Day)**<br><br>2.1 Monitor vital signs (blood pressure, pulse).<br><br>2.2 Perform surgery using the designated device according to randomization results, recording anesthesia method, device model, and device performance evaluation.<br><br>2.3 Transesophageal echocardiography (TEE) or transthoracic echocardiography (TTE) evaluation as needed.<br><br>2.4 Record intraoperative complications and adverse events.<br><br>2.5 Record intraoperative antiplatelet or anticoagulant medications.<br><br>**3 Clinical Evaluation at Discharge or 30 Days (±7 Days) Postoperatively (Whichever Comes First)**<br><br>3.1 Monitor vital signs (blood pressure, pulse).<br><br>3.2 NYHA functional class.<br><br>3.3 ECG, echocardiography (TTE).<br><br>3.4 Laboratory tests: Complete blood count, blood biochemistry.<br><br>3.5 Record adverse events.<br><br>3.6 Record antiplatelet or anticoagulant medications.<br><br>**4 Clinical Evaluation at 3 Months (±15 Days) Postoperatively**<br><br>4.1 NYHA functional class.

	4.2 ECG, echocardiography (TTE).<br>4.3 Record adverse events.<br>4.4 Record antiplatelet or anticoagulant medications.<br>**5 Clinical Evaluation at 6 Months (±30 Days) Postoperatively**<br>5.1 NYHA functional class.<br>5.2 ECG, echocardiography (TTE).<br>5.3 Record adverse events.<br>5.4 Record antiplatelet or anticoagulant medications.<br>**7.2.6 Clinical Evaluation at 12 Months (±30 Days) Postoperatively**<br>6.1 NYHA functional class.<br>6.2 ECG, echocardiography (TTE).<br>6.3 Record adverse events.<br>6.4 Record antiplatelet or anticoagulant medications.<br>**7 Clinical Evaluation at 2, 3, 4, and 5 Years (±30 Days) Postoperatively**<br>7.1 Telephone follow-up.<br>7.2 Record adverse events.

Statistical Methods:

This trial plans to enroll 164 patients, who will be randomly assigned to either the experimental group or the control group in a 1:1 ratio, with 82 cases in each group.

The primary endpoint of this clinical trial is the success rate of valve repair at 6 months postoperatively, defined as no severe regurgitation and an increase in annular diameter of ≤15% based on echocardiographic evaluation. According to literature reports and clinical experience, the expected success rate of valve repair in the control group at 6 months postoperatively is 95%. Assuming that the investigational medical device can achieve comparable efficacy, the clinically accepted non-inferiority margin is set at -10%. With a one-sided significance level of 0.025 and a power of 80%, the sample size was estimated using PASS 14.0 software, indicating that 75 cases are required for each group. Considering potential dropouts during the study, the plan is to enroll 82 cases per group, totaling 164 cases.

The trial results will be determined based on the difference in the primary efficacy endpoint between the experimental group and the control group. If the lower limit of the 95% confidence interval for the difference in success rates between the experimental group and the control group is

greater than -10% (the predefined non-inferiority margin), the non-inferiority conclusion will be established. Otherwise, the non-inferiority conclusion will not be supported.

Protocol Text

1 .Sponsor Information

**1.1 Sponsor Name:**	Permed Biomedical Engineering Co., Ltd
**1.2 Sponsor Address:**	No.7 Warehouse, No.9 Tianfu Street, Daxing District, Beijing
**1.3 Contact information:**	Legal representative: Bian Yi<br>Project Contact Person: Yufang Sun<br>Tel: 180 1900 8461<br>Email: yufang.sun@cardimed.com.cn
**1.4 Qualification of Sponsor**	Business License<br>Unified Social Credit Code: 91110115102035909D

2 . List of all clinical trial institutions and investigators for multicenter clinical trial:

Leading site: The Affiliated Drum Tower Hospital of Nanjing University Medical School
Coordinating-investigator: Prof. Dr. Dongjin Wang

Serial Number	Name of clinical trial institution	Investigator	Contact number
01	The Affiliated Drum Tower Hospital of Nanjing University Medical School	Wang Dongjin	13915980346
02	The First Hospital of China Medical University	Gu Tianxiang	13998891869
03	The Second Hospital of Jilin University	Liu Kexiang	13943136040
04	Renji Hospital Shanghai Jiao Tong University School of Medicine	Xue Loose	13501754558
05	The Second Affiliated Hospital of Nanchang University	Jianjun Xu	13907913526
06	The First Affiliated Hospital of Zhengzhou University	Joe Chenhui	13513893267
07	Xiangya Hospital of Central South University	Luofan 's ink stone	13973129106
08	Tongji Hospital, Tongji Medical College of HUST	Wei Xiang	13995525956
09	First Affiliated Hospital of Kunming Medical University	Pottery Jay	13708885999

10	Affiliated Cardiovascular Hospital of Xiamen University	Wu Xijie	13799959630
11	The Second Affiliated Hospital of Harbin Medical University	Field sea	13904501826
12	Dalian Central Hospital	He Xuezhi	13352282030
13	Sichuan Provincial People 's Hospital, Sichuan Academy of Medical Sciences	Huang Keli	18981838658
14	The First Affiliated Hospital of Jinan University (Guangzhou Overseas Chinese Hospital)	Zhang Xiaoshen	13802830960
15	Nanfang Hospital of Southern Medical University	Shaoyi Zheng	13602836788
16	The First People 's Hospital of Jining	Zhang Shen	18678769807
17	Chongqing People 's Hospital	Chen Haemophilus	13883800600
18	The First Affiliated Hospital of University of South China	Feng Yaoguang	13100261988
19	Shenzhen People 's Hospital (The Second Clinical Medical College of Jinan University)	Zheng Qijun	18025385866
20	Sun Yat-sen Memorial Hospital, Sun Yat-sen University	Junmeng Zheng	13902828148

Note: In case of slow enrollment, changes in policies and regulations, force majeure and other factors, after ethical approval by the site, the number of clinical sites may be increased or decreased appropriately, up to 20.

3. Purpose and content of clinical trial

3.1Object

To evaluate the safety and efficacy of cardiac annuloplasty ring developed by Permed Biomedical Engineering Co., Ltd for the treatment of tricuspid insufficiency.

3.2Content

This trial is designed as a prospective, multi-center, randomized controlled study to evaluate the safety and efficacy of a cardiac annuloplasty ring developed by Permed Biomedical Engineering Co., Ltd. in patients with tricuspid insufficiency. According to the inclusion and exclusion criteria of this trial, at least 164 subjects will be enrolled. All subjects will be randomly divided into the test group or the control group. The test group will use the cardiac annuloplasty ring developed by Permed

Biomedical Engineering Co., Ltd., while the control group will use the cardiac annuloplasty ring manufactured by Edwards Lifesciences (Shanghai) Medical Products Co., Ltd., for tricuspid valve annuloplasty. Imaging and/or clinical follow-up will be performed intraoperatively, before discharge or 30 days ± 7 days postoperatively, 3 months ± 15 days postoperatively, 6 months ± 30 days postoperatively, and 12 months ± 30 days postoperatively. The primary study endpoint is the success rate of annuloplasty ring repair at 6 months after surgery, defined as the absence of severe regurgitation and ≤ 15% increase in radial line value (based on a comprehensive determination of the degree of regurgitation and increase in radial line value) as evaluated by echocardiography. Secondary effectiveness evaluation endpoints include the degree of regurgitation indicated by cardiac ultrasound before discharge or at 30 days after operation (whichever comes first), and at 3, 6, and 12 months after operation, changes in echocardiographic parameters (right atrium size, right ventricular size, diameter value, width of the narrowest part of the regurgitant bundle, and reverse flow area), NYHA functional classification, and freedom from reoperation of the tricuspid valve due to tricuspid insufficiency. Performance evaluation of the test device includes smooth needle insertion and ease of use of the ring holding device and technical success rate of the operation (before leaving the operating room), defined as no intraoperative death, successful ring implantation, and holding Successful removal of the device without emergent procedure or secondary intervention related to the investigational device; Safety endpoints included the incidence of serious adverse events (such as cardiovascular death related to the investigational device, major bleeding, major damage to cardiac or other body structures, infective endocarditis, thromboembolic events), all-cause death, cardiovascular death related to the investigational device, major bleeding related to the investigational device, major damage to cardiac or other body structures related to the investigational device, infective endocarditis related to the investigational device, thromboembolic events related to the investigational device, device defects, and other adverse events occurring before discharge or within 30 days after the procedure, whichever came first. Follow-up assessments included clinical symptoms and signs, laboratory examinations, ECG, color Doppler ultrasound, and recording of adverse events, all aimed at verifying the safety and effectiveness of the investigational annuloplasty ring.

It is planned to issue a summary report to the China Food and Drug Administration after the 6-month follow-up of the subjects is completed. A one-year follow-up report will be issued after the 12-month follow-up is completed. Additionally, follow-up assessments will be performed at 2 years ± 30 days, 3 years ± 30 days, 4 years ± 30 days, and 5 years ± 30 days postoperatively to evaluate the long-term safety of the investigational product.

4. Background data of clinical trials

4.1 Study rationale

Tricuspid insufficiency refers to the inability of the tricuspid valve to close normally during right ventricular systole due to leaflet thickening, poor coaptation, or annular dilation of the tricuspid valve. Tricuspid valve insufficiency is one of the common valvular heart diseases, with an incidence of about 0.8% in the general population and 35% in patients with heart failure. Based on the structure of the tricuspid valve, it can be divided into functional and organic types. Organic tricuspid insufficiency is relatively rare (about 25%), while functional tricuspid insufficiency (FTI) is the most common, accounting for about 75%. FTI is caused by tricuspid annular dilation due to pulmonary hypertension resulting from left-sided heart valve disease or left-to-right shunt congenital heart disease [1, 2]. Rheumatic left-sided valvular disease and congenital heart disease are the leading causes of functional tricuspid insufficiency, with rheumatic mitral valve disease accounting for approximately 74% of all cases [3].

Functional tricuspid insufficiency is a common dysfunction of the tricuspid valve. Because the right ventricular wall is highly sensitive to volume overload and the right ventricle becomes thinner, it can lead to increased volume load. Right ventricular dysfunction and increased afterload can induce functional tricuspid regurgitation (FTR) [4]. Since FTR is relatively common, not only in patients with structural and functional heart disease but also in healthy individuals, mild to moderate tricuspid regurgitation accounts for 25% of functional tricuspid regurgitation cases. Due to the low degree of regurgitation, there is often a lack of obvious symptoms and signs, and it has long been neglected [5, 6]. Recent studies have found that even mild to moderate tricuspid regurgitation can affect disease prognosis.

Both the American College of Cardiology (ACC)/American Heart Association (AHC) and the European Society of Cardiology (ESC)/European Association for Cardiothoracic Surgery (EACTS) recommend tricuspid valve surgery in patients with severe tricuspid regurgitation and those with significant tricuspid annular enlargement or significant pulmonary hypertension at the same time as left heart valve surgery [7, 8] . Many domestic scholars also believe that FTR lesions should be actively treated with surgery, surgery is mostly performed at the same time when left heart surgery is performed, the main treatment is tricuspid valvuloplasty, and artificial annulus implantation is the best.

In order to treat functional tricuspid insufficiency, repair tricuspid valve function, and improve tricuspid regurgitation, Permed Biomedical Engineering Co., Ltd. successfully developed a cardiac annuloplasty ring. This trial is being conducted to verify the safety and effectiveness of this device in clinical practice.

4.2 Study background

Tricuspid insufficiency refers to the right ventricular systolic tricuspid valve unable to close normally due to leaflet thickening, poor differentiation, or annular dilatation of the tricuspid valve. Tricuspid valve insufficiency is one of the common valvular heart diseases. According to the structure of tricuspid valve, it can be divided into functional and organic types. Organic tricuspid insufficiency is mostly the result of fibrosis and curling of valve and chordae tendineae scar caused by rheumatic fever after tricuspid rheumatic endocarditis; while functional tricuspid insufficiency is due to increased right atrial pressure caused by various reasons (such as left heart system valvular disease), pulmonary hypertension caused by reverse conduction, increased right ventricular afterload, and finally leads to right ventricular cavity deformation, tricuspid annular enlargement, displacement of subvalvular structure, resulting in secondary tricuspid regurgitation [9] .

The tricuspid valve as a whole consists of three leaflets (anterior, posterior, septal), chordae tendineae, two separate papillary muscles, annulus fibrosus of the tricuspid valve, myocardium of the right atrium and right ventricle. Good valve function relies on the integrity and coordination of the various

components. Anatomical studies have shown that the enlargement of the tricuspid annulus is unequal among the three leaflets, with significant posterior valve involvement, which can enlarge by about 80% compared with normal, the anterior annulus is smaller, which can enlarge by about 40% compared with normal, and the septal annulus is only enlarged by 10% [10] ; based on these characteristics, the measurement of the tricuspid annulus depends on the size of the leaflets. The malleability of the tricuspid valve is very good and can change significantly at different pressure/load volumes, and even during atrial systole, the circumference of the annulus is reduced by approximately 19% (30% area reduction) [11] , So, in the case of severe left heart disease with left atrial pressure overload, the pressure load is inversely transmitted, causing increased pulmonary venous pressure and pulmonary circulation congestion. Long-term pulmonary congestion leads to fibrosis of the pulmonary interstitial tissue and pulmonary vasculature, which in turn causes chronic pulmonary vascular remodeling and pulmonary artery constriction. This results in pulmonary hypertension and the formation of right ventricular pressure overload. These changes then cause dilation of the tricuspid valve, flattening and rounding of the tricuspid annulus, and alteration of the spatial relationships among the leaflets, annulus, chordae tendineae, papillary muscles, and other important tissue structures. This leads to deviation of the traction forces acting on the leaflets and asymmetric contraction of the annulus, ultimately preventing normal coaptation of the tricuspid leaflets and resulting in tricuspid regurgitation.

The 2008 ACC/AHA guidelines for valvular heart disease state [12] that patients with severe tricuspid regurgitation should undergo concomitant tricuspid valvuloplasty (recommendation level I) during mitral valve surgery; while for patients with mild to moderate tricuspid regurgitation, pulmonary hypertension or tricuspid annular dilatation are also recommended to perform mitral valve surgery

Stage management of tricuspid valve (but recommended grade II b). Valvular heart disease guidelines issued by the ESC/EACTS in 2012 were significantly more aggressive in recommendation levels. Simultaneous treatment of the tricuspid valve (II a) is recommended during left-heart valve

surgery in patients with small or moderate amount of secondary tricuspid regurgitation as long as there is dilatation of the tricuspid annulus (≥ 40 mm or 21 mm/m 2); for severe isolated primary tricuspid regurgitation, surgical treatment is recommended in grade I as long as clinical symptoms occur, regardless of severe right ventricular dysfunction [13] . New Heart released by ACC/AHA in 2014

Visceral valvular disease guidelines are very similar to the 2012 ESC/EACTS guidelines, and an additional one is added to this

— Patients with moderate functional tricuspid regurgitation and pulmonary hypertension should be considered for tricuspid valvuloplasty during left heart surgery (recommendation grade II a) [14] . Due to the complexity of tricuspid valve complex, its lesions are affected by many complex dynamic factors, and show a progressively increasing development process, resulting in that its degree of regurgitation and right ventricular functional status are difficult to accurately assess, and the current surgical indications for the treatment of functional tricuspid regurgitation are more and more radical. Preoperative determination of the severity of tricuspid valve disease depends on the regurgitation of the tricuspid valve on the one hand, and more importantly on the size of the right ventricular cavity, the degree of dilatation of the tricuspid annulus, and the degree of corresponding pathological changes of the tricuspid leaflets . [15]

For the treatment of tricuspid valve disease, there are two kinds of surgical procedures: tricuspid valve replacement and tricuspid valvuloplasty in clinical practice; while tricuspid valvuloplasty is divided into two categories: artificial annular implantation and linear annuloplasty. Tricuspid valve replacement is rarely used in tricuspid valve management and is generally more performed in patients with severe tricuspid stenosis or infective endocarditis. Although tricuspid valve replacement is more effective than tricuspid valvuloplasty in correcting tricuspid regurgitation and preventing its recurrence in the early and late postoperative period, there is no significant difference in 10-year survival rate or even lower than tricuspid valvuloplasty [16, 17] .

De Vega tricuspid annuloplasty is a commonly used linear annuloplasty

technique and one of the important methods to correct tricuspid regurgitation. Mainly suturing the enlarged anterior and posterior annulus, the annulus attached to the free wall of the right ventricle is reduced so that the orifice can accommodate only two to two finger half-widths, thereby correcting tricuspid insufficiency. At present, the mainstream view at home and abroad believes that this plasty method only uses suture annular suture, which is a temporary support method, and the surgical effect is not very clear. West China Hospital has compared 148 patients who underwent tricuspid valvuloplasty and prosthetic annuloplasty in their cardiac surgery department, and De Vega valvuloplasty is not ideal for correcting tricuspid regurgitation[18]. However, some scholars, such as Parolari et al., summarized a meta-analysis published in 2017 that found no significant difference in long-term (up to 15 years) survival rates between the ring-forming and linear-forming methods. [19] In 2016, Shinn from the MayoClinic and his colleagues analyzed 479 patients who underwent tricuspid valve repair concurrently with mitral valve surgery between 1995 and 2010. The primary cause was degenerative diseases, including rheumatic and ischemic conditions. The tricuspid valve repair methods included artificial soft ring implantation (54%), Key repair, or De vega repair (46%). The overall five-year survival rate was 69%, and the ten-year survival rate was 44%; the study also noted that there was no significant difference in late survival rates or the durability of tricuspid valve repair between artificial valves and De Vega repair [20].

Kays Procedure:In severe tricuspid valve functional insufficiency, the posterior leaflet exhibits the greatest dilation during annular expansion. The chordae tendineae attached to the posterior leaflet and the traction forces from the papillary muscles are relatively weak, making this leaflet the primary site of insufficiency. The Kays procedure addresses this by reconstructing the tricuspid valve to close the posterior annulus, thereby fully utilizing the closing function of the anterior leaflet and eliminating insufficiency. This technique is particularly suitable for patients with significantly dilated annuli [21]. While the Kays procedure is technically straightforward, its theoretical drawback is that it does not address the dilation of the remaining parts of the right ventricular free wall in the tricuspid annulus. Consequently, postoperative residual

regurgitation rates and recurrence rates are relatively high, limiting its use in clinical practice.

Artificial valve implantation is the preferred surgical procedure for treating functional tricuspid regurgitation in most cardiac centers across China. Artificial valves are categorized into soft rings, hard rings, semi-soft rings, and three-dimensional artificial valves. While hard rings can effectively stabilize the valve ring shape, prevent tricuspid annular dilation, and reduce tricuspid regurgitation, they limit the contractility and compliance of the tricuspid annulus, thereby affecting the normal function of the right ventricle. Soft rings, although well-suited to the dynamic changes of the tricuspid valve, tend to deform easily and have significantly reduced effectiveness in controlling regurgitation under prolonged pulmonary artery and right ventricular hypertension.

Carpentier Artificial Rigid Annulus Repair: In 1974, Carpentier [22] first introduced the use of a rigid prosthesis for annulus reconstruction. This method involves using a 20-gauge Ticron double-ended needle with a pledget to perform interrupted mattress sutures along the anterior and posterior septa, from the junction of the anterior to the posterior septa. The needle spacing for the annulus is slightly wider, while that for the prosthesis is slightly narrower, allowing effective annular contraction and fixation. The advantages of this technique include uniform stress distribution throughout the annulus during contraction, preservation of the normal physiological shape of the tricuspid valve while maximizing correction of an overextended annulus, and precise alignment of the prosthesis gap with the septal valve area to prevent damage to the chordae tendineae. Compared to the De Vega repair, postoperative recurrent regurgitation is less common. However, the rigid annulus lacks compliance and is non-adjustable in size. This limits the dynamic movement of the tricuspid annulus during the cardiac cycle, potentially affecting right ventricular function and leading to relative tricuspid stenosis during diastole. It also increases the risk of postoperative thrombosis and annulus rupture.

The Duran elastic ring was introduced by Duran [23] in 1976. It is an oval soft ring without a notch and is shaped into a square configuration. The surgical

technique is similar to that of the C-E ring. When suturing the septal annulus, the needle should first enter from the atrial surface, then enter the ventricular surface, re-enter at a distance of 2 mm, and finally exit from the atrial surface to avoid damaging the conduction bundle within the Koch triangle. Duran et al. followed up on 91 patients who had this stent implanted, reporting a postoperative thrombosis rate of 4.8% and no cases of annular tearing [24]. The advantage of this stent is its ability to maintain the flexibility of both the anterior and posterior annuli without sutures, simplifying the procedure. Compared to the C-E ring, its physiological movement more closely matches the structure of the tricuspid valve, reducing the risk of relative tricuspid stenosis during diastole.

Cosgrove-Edwards Artificial Soft Ring Valvuloplasty: Initially applied to the posterior leaflet of the mitral valve, the Cosgrove-Edwards artificial soft ring valvuloplasty utilizes a C-shaped elastic soft ring. Due to its softness and inherent elasticity, Cosgrove [25] adapted this technique for tricuspid valve valvuloplasty. The surgical technique is similar to that of Carpentier rings, but it does not use pledgets during suturing. After removing the support, the C-shaped ring bends at the anterior-posterior junction under elastic stress, reinforcing the valve ring at its most dilatable point. Gati et al. [26] reported that between 1998 and 2002, they used Cosgrove-Edwards MC3 rings to treat 35 patients with preoperative regurgitation >2+. Postoperatively, regurgitation was controlled at 1+. The patients had cardiac function (NYHA) grades I-II, and after three years of follow-up, 96.8% had mild or less severe regurgitation.The primary advantage of this ring is its ability to change size and shape dynamically with the cardiac cycle, adapting to the movement of the natural valve annulus. This feature maintains right ventricular function and better conforms to normal physiological conditions and anatomical compliance. Additionally, the wireless design of the septal valve ring avoids damage to the conduction bundle located anterior to the septum. Currently, this technique is widely used in clinical practice.

Edwards MC3 Valve Ring Reshaping: In three-dimensional space, the dilated tricuspid valve has an anatomical structure resembling a saddle, with a shallower base near the aorta and a deeper portion at the septum. Previous methods of reshaping were based on two-dimensional approaches. McCarthy [27] designed a new rigid C-shaped three-dimensional reshaping ring. Compared to the Carpentier ring, its primary

advantage is that the valve ring is pre-molded into a saddle shape, which more closely conforms to the three-dimensional anatomy of the tricuspid valve annulus. Additionally, after titanium alloy treatment, the reshaping ring maintains a certain degree of elasticity, reducing suture tension and ensuring more precise valve reshaping.The postoperative risk of annular rupture and damage is low, significantly reducing the incidence of regurgitation. Filsoufil[28] et al. applied MC3 ring commissurotomy to 75 patients between 2002- 2004, with a mortality rate of 53% (4 cases) in early follow-up,averaging 16 months. The degree of regurgitation decreased from preoperative (3.1±0.9) to (0.3±0.5), with no complications such as thrombosis, artificial annulus rupture, or infective endocarditis, demonstrating satisfactory early outcomes. Fukuda et al. used MC3 commissurotomy for 136 patients, with mid-to-long-term follow-up results showing a reduction in regurgitation percentage from 40.0±16.6% to 11.8±10.3%, along with decreased right ventricular systolic pressure and right heart volume. Currently, due to the lack of long-term follow-up data and prospective clinical comparisons, it cannot be definitively determined that MC3 rings are superior to other commissurotomy rings, but they have shown early and mid-term benefits.The follow-up results show that this shaped ring has application prospect.

Due to the high cost of artificial valves, autologous pericardial patches have been used in tricuspid valve repair to reinforce the anterior and posterior annuli, thereby preventing secondary dilation at the septal base and achieving significant results. The pericardial patch is first soaked in glutaraldehyde for 15 minutes, then rinsed with saline and cut into suitable strips for later use. Next, 3-0 Prolene sutures are used to perform interrupted horizontal mattress sutures along the tricuspid annulus, from the anterior septal commissure to the posterior septal commissure, with approximately 5-6 stitches. Each stitch passes through a pericardial strip, with the stitch spacing on the annulus being greater than that on the pericardial strips. Knots are tied sequentially, starting from the posterior septal commissure side, while observing for regurgitation during suturing to ensure no obvious regurgitation under direct vision. The length of the pericardial patch and the number of stitches are determined based on the degree of annular dilation and valve closure.

In a follow-up study involving 59 patients who underwent pericardial patch repair, De Lazerda et al. [30] reported a 5-year survival rate of 98.4%, a 100% rate of avoiding reoperation, a mild regurgitation rate of 67.2%, a moderate regurgitation rate

of 31%, and a severe regurgitation rate of 1.8%. The advantages of this technique include reducing and reinforcing the annulus while maintaining its compliance, preventing the annulus from being cut by sutures, and not affecting the leaflet area. Additionally, autologous pericardial material is readily available, with no postoperative rejection and favorable growth of endocardial cells. Postoperatively, no further intervention is required. Anticoagulation therapy avoids complications such as thrombosis, embolism, and bleeding. However, a disadvantage is that pericardial tissue cannot continue to grow, potentially facing issues such as calcification, durability, and related hemodynamic characteristics. Long-term efficacy remains to be further observed.

Based on current literature reports and practical experiences, the active management of functional tricuspid regurgitation has gained widespread recognition. However, there is still some controversy regarding the choice of surgical methods, which remains a challenging issue in cardiac surgery. The selection of tricuspid valve repair techniques, concurrent mitral valve intervention, and correction of related risk factors still require attention and further research [31-37].

The heart valve stent independently developed by Permed Biomedical Engineering Co., Ltd has completed testing at the Jinan Medical Device Quality Supervision and Inspection Center of the National Medical Products Administration, obtaining a qualified inspection report. Additionally, animal experiments were conducted at the Animal Experiment Center of Fuwai Hospital, Chinese Academy of Medical Sciences, achieving the expected experimental objectives. The results met the requirements for biological reproducibility and complied with GB12279-2008 "Artificial Heart Valves for Cardiovascular Implants." This experiment confirmed that the Inclusive heart valve stent (tricuspid stent) can safely integrate with the native tricuspid valve structure after implantation in small-tailed cold sheep, performing its normal functions. Its safety and effectiveness are comparable to those of the Edwards MC3 Rings Based on these findings, the feasibility of conducting a prospective, multicenter, randomized controlled clinical trial to evaluate the safety and effectiveness of this product in tricuspid valve repair is established.

5.Product Features, Structural Composition, Working Principle, Mechanism of Action, and Testing Scope

5.1 Product Features

The Annuloplasty Ring for cardiac valve repair features a nitinol tube as its base. The ring adopts a three-dimensional oval shape, conforming to the physiological structure of the tricuspid annulus. The ring is pre-mounted on a holder for ease of implantation. Clinically, it is used to correct and remodel pathological annuli, maintaining optimal leaflet coaptation.

5.2 Structural Composition, Working Principle, and Mechanism of Action

The Annuloplasty Ring comprises the following layers:

Base layer: A closed-loop nitinol tube connected with nitinol wires.Intermediate layer: A silicone layer with a suture border, filled with long-term implantable silicone between the base and intermediate layers.Outer layer: A biocompatible polyester fabric.The Annuloplasty Ring is designed for tricuspid valve repair. Its three-dimensional oval shape matches the natural tricuspid valve orifice. The ring comes pre-attached to a sterile, single-use holder for simplified implantation. The holder and sizing device are packaged separately as non-sterile components, requiring cleaning and steam sterilization before reuse.

prosthetic ring

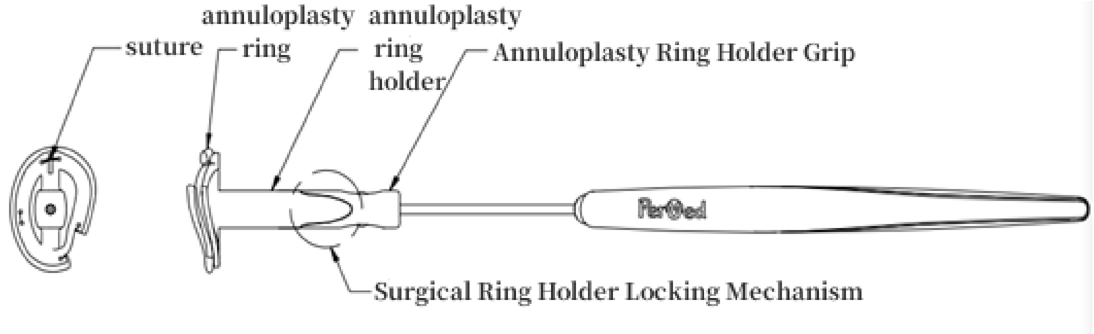

Figure 1: Schematic of Annuloplasty Ring Components

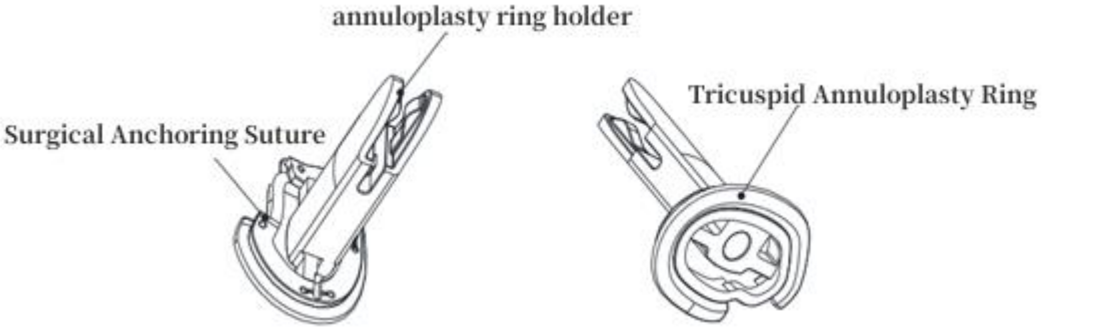

Figure 2: Annuloplasty Ring Schematic

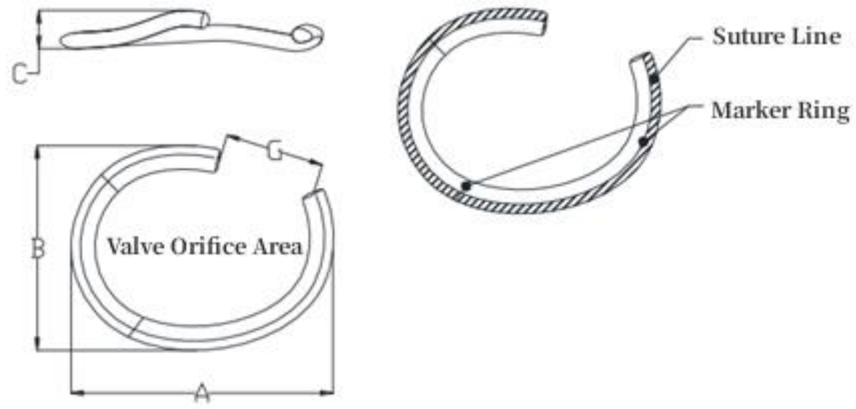

Figure 3: Annuloplasty Ring Schematic

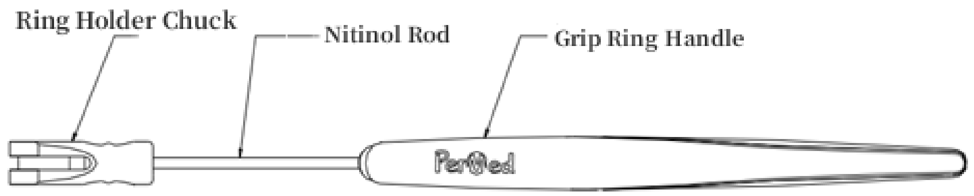

Figure 4: Holder Handle Schematic

5.3 Testing Scope

Applicable to patients with tricuspid regurgitation who are eligible for Annuloplasty Ring implantation.

6. Indications, Contraindications, Precautions

6.1 Indications

The Annuloplasty Ring is indicated for:Correction of annular dilation.Enhancement of leaflet coaptation and annular stabilization.Prevention of further annular expansion.

6.2 Contraindications

6.2.1 Pediatric patients with potential somatic growth affecting valve area.

6.2.2 Active bacterial endocarditis during device implantation.

6.3 Precautions

6.3.1 Surgeons must receive procedural training prior to clinical use.

6.3.2 The sizing device and holder handle must be cleaned and steam-sterilized for reuse.

6.3.3 Ring sizing must follow precise annular measurements using the manufacturer-provided sizing device.Do not use the holder as a sizing tool.
6.3.4 Avoid using sharp needles or metal forceps on the ring during implantation to prevent damage.
6.3.5 Sutures must not penetrate atrial tissue or coronary arteries (left circumflex or right coronary artery) to avoid conduction system injury.
6.3.6 Remove the holder before closing the surgical site.If retained, the holder may cause severe injury or death. Use intraoperative X-ray to confirm removal if necessary.
6.3.7 Postoperative anticoagulation therapy is recommended. Prophylactic antibiotics are advised for patients undergoing dental or invasive procedures with bacteremia risk.

6.4 Storage and Transportation

Storage: Store at room temperature in a dry, uncontaminated environment. Do not use expired products.
Transportation: Avoid impact, compression, or exposure to moisture. Discard the product if damaged during transit.

6.5 Complications

6.5.1 Severe complications, including death, may arise from adverse reactions to the implant, necessitating reoperation or device replacement.
6.5.2 Surgical complications include, but are not limited to:
Residual or recurrent valve regurgitation: The valve may continue to leak or start leaking again after surgery.
Valve stenosis: The valve may become narrowed, restricting blood flow.
Thrombosis: Blood clots may form, potentially blocking blood flow.
Hemolysis: Red blood cells may break down abnormally.
Atrioventricular (AV) block: The electrical signals in the heart may be disrupted, affecting heart rhythm.
Low cardiac output: The heart may not pump enough blood to meet the body’s needs.
Right heart failure: The right side of the heart may fail to function properly.
Degenerative changes or dysfunction of the natural valve: The patient’s own valve may deteriorate or malfunction due to worsening disease.
Endocarditis: An infection of the heart’s inner lining or valves.

Incomplete repair of the valve or subvalvular structures: The repair may not fully correct the valve or its supporting structures.

Coronary artery injury caused by sutures: The stitches used during surgery may damage the coronary arteries.

Complications from prolonged cardiopulmonary bypass or aortic cross-clamping: Extended use of the heart-lung machine or restricted blood flow during surgery may cause complications.

Inadequate myocardial protection: The heart muscle may not be sufficiently protected during surgery, leading to damage.

Tearing of the polyester fabric due to sharp needles: The fabric used in the device may tear if sharp needles are used improperly.

Suture breakage due to improper placement in the Annuloplasty Ring: Stitches may break if they are not placed correctly in the ring.

Bleeding caused by anticoagulation therapy: Blood-thinning medications may lead to excessive bleeding.

Local or systemic infections: Infections may occur at the surgical site or spread throughout the body.

6.5.3 Device-related complications include, but are not limited to:

Residual or recurrent valve regurgitation: The valve may continue to leak or start leaking again after surgery.

Valve stenosis: The valve may become narrowed, restricting blood flow.

Thrombosis: Blood clots may form, potentially blocking blood flow.

Hemolysis: Red blood cells may break down abnormally.

Atrioventricular (AV) block: The electrical signals in the heart may be disrupted, affecting heart rhythm.

Low cardiac output: The heart may not pump enough blood to meet the body’s needs.

Right heart failure: The right side of the heart may fail to function properly.

Degenerative changes or dysfunction of the natural valve: The patient’s own valve may deteriorate or malfunction due to worsening disease.

Endocarditis: An infection of the heart’s inner lining or valves.

Incomplete repair of the valve or subvalvular structures: The repair may not fully correct the valve or its supporting structures.

Coronary artery injury caused by sutures: The stitches used during surgery may damage the coronary arteries.

Complications from prolonged cardiopulmonary bypass or aortic cross-clamping: Extended use of the heart-lung machine or restricted blood flow during surgery may cause complications.

Inadequate myocardial protection: The heart muscle may not be sufficiently protected during surgery, leading to damage.

Tearing of the polyester fabric due to sharp needles: The fabric used in the device may tear if sharp needles are used improperly.

Suture breakage due to improper placement in the Annuloplasty Ring: Stitches may break if they are not placed correctly in the ring.

Bleeding caused by anticoagulation therapy: Blood-thinning medications may lead to excessive bleeding.

Local or systemic infections: Infections may occur at the surgical site or spread throughout the body.

6.5.4 Report all complications to PerMed immediately: Tel: +86 10 6125 2408; Fax: +86 10 6125 2476.

7. Overall Trial Design

7.1 Trial Design

7.1.1 Objective

To evaluate the safety and efficacy of the Annuloplasty Ring, developed by PerMed Bio-Medical Engineering Co., Ltd., Beijing, for treating tricuspid regurgitation.

7.1.2 This trial is designed as a prospective, multicenter, randomized controlled clinical trial to evaluate the safety and efficacy of the Annuloplasty Ring for tricuspid regurgitation.

Rationale:

7.1.2.1 Prospective, Multi-Center Design

Prospective: This means the trial plan is designed and finalized before the study begins. This approach is standard for pre-market clinical trials of medical devices, as it ensures a clear and structured process for evaluating safety and effectiveness.

Multi-Center: The trial will be conducted across more than ten clinical sites nationwide. By involving multiple centers, the study can include a more diverse and representative group of participants. This reduces the risk of bias that might occur if

the trial were conducted at a single center, making the results more reliable and credible.

7.1.2.2 Rationale for Randomized Controlled Design

The randomized controlled trial (RCT) design is chosen because it provides a high level of reliable evidence in clinical research. In this trial, participants who meet the inclusion criteria will be randomly assigned to one of two groups:

Test Group: Participants will receive the trial Annuloplasty Ring.

Control Group: Participants will receive a commercially available Annuloplasty Ring (manufactured by Edwards, which has been approved by the National Medical Products Administration (NMPA). This control device is widely used in clinical practice and has a well-established efficacy profile. Random assignment ensures balanced and comparable groups, minimizing bias and enhancing the reliability of the results.

Dynamic Randomization: The trial uses a dynamic randomization method, taking into account two key factors:

Study Center: Ensures balanced distribution across multiple sites.

Severity of Regurgitation: Participants are stratified based on the degree of valve leakage (mild, moderate, or severe).

Central Randomization System: The trial employs a Clinical Trial Central Randomization System (DAS for IWRS) to allocate random numbers, ensuring fairness and transparency in group assignment.

7.1.2.3 Rationale for Selecting the Primary Endpoint：

The primary goal of the Annuloplasty Ring is to repair and reshape the diseased valve annulus, ensuring proper leaflet coaptation (the area where the valve leaflets meet). For patients with tricuspid regurgitation (leaky valve), the main evaluation metric after annulus repair is the success rate of the Annuloplasty Ring at 6 months post-surgery. Definition of Repair Success: No Severe Regurgitation: Postoperative echocardiography (heart ultrasound) must show no severe valve leakage. Annular Dilation ≤15%: The increase in the annular size (measured by echocardiography) must be 15% or less compared to preoperative measurements.

Secondary Efficacy Endpoints

The secondary endpoints for evaluating effectiveness include the following assessments at discharge or 30 days post-surgery (whichever comes first), and at 3 months, 6 months, and 12 months post-surgery:

Degree of Valve Regurgitation: Assessed by echocardiography (heart ultrasound) to measure the severity of valve leakage.

Echocardiographic Parameters: Changes in the following measurements will be evaluated:

Size of the right atrium and right ventricle.

Annular dimensions (diameter).

Width of the narrowest part of the regurgitant jet.

Area of reverse blood flow.

NYHA Functional Class: A measure of heart failure symptoms and functional capacity, classified as Class I (no symptoms) to Class IV (severe symptoms).

Freedom from Reoperation: The rate of patients who do not require repeat surgery for tricuspid regurgitation.

Device Performance Evaluation:

Suture Needle Ease of Use: How smoothly the needle passes through the Annuloplasty Ring during implantation.

Holder Device Usability: How user-friendly the ring holder is during the procedure.

Surgical Technical Success:

Defined as the following criteria being met before leaving the operating room:

No intraoperative death.

Successful implantation of the Annuloplasty Ring.

Successful removal of the ring holder.

No need for emergency surgery or additional interventions related to the investigational device.

Safety Endpoints:

Safety will be assessed at discharge or 30 days post-surgery (whichever comes first), and at 3 months, 6 months, and 12 months post-surgery. The following will be evaluated:

Serious Adverse Events (SAEs): Including:

Cardiovascular-related deaths linked to the device.

Major bleeding linked to the device.
Severe damage to the heart or other body structures linked to the device.
Infective endocarditis (heart infection) linked to the device.
Thromboembolic events (blood clots) linked to the device.
All-Cause Mortality: Death from any cause during the study period.
Device-Related Adverse Events: Any complications or issues directly related to the device.

Device Defects: Any malfunctions or failures of the device.

Follow-up content includes clinical symptoms and signs, laboratory examinations, ECG, CDFI, recording adverse events, etc., to verify the safety and effectiveness of the trial cardic Annuloplasty Ring.

7.1.3 Measures to Reduce and Avoid Bias

To ensure the reliability and validity of the trial results, bias will be minimized through the following three key strategies:

Randomization Design

The trial is designed as a randomized controlled trial (RCT), which is the gold standard for minimizing bias in clinical research. After a participant (or their legal guardian) signs the informed consent form and meets the eligibility criteria, they will be randomly assigned to either the test group or the control group using a centralized randomization system. This approach ensures that the two groups are balanced and comparable, reducing the risk of bias and improving the reliability of the results.

Investigator Training

Before the trial begins, the sponsor and clinical monitors will work with the principal investigators at each trial site to provide comprehensive training on the trial protocol.

Investigators will be trained to: Understand and properly use the investigational product. Stay informed about any new information or updates related to the product during the trial. This ensures that all investigators follow the same procedures and maintain consistency across all trial sites.

Clinical Trial Monitoring

A monitoring plan will be established, and qualified monitors appointed by the sponsor will conduct regular on-site visits to the trial hospitals. The monitors will:

Ensure that the trial protocol is strictly followed at all sites. Verify that the data recorded in the electronic Case Report Forms (eCRFs) match the original source documents (e.g., medical records).This oversight helps maintain the integrity of the trial data and ensures compliance with regulatory and ethical standards.

7.1.4 Investigational and Control Devices

During surgery, the measurement of the tricuspid complex septal base length serves as a criterion for selecting the appropriate annulus size. Based on the results of randomization, the tricuspid Annuloplasty Ring from the appropriate test or control group is then selected.

Investigational Device: Annuloplasty Ring (Beijing Permed Biomedical Engineering Co..Ltd) /(sizes 26、28、30、32、34、36).

Control Device: Edwards MC3 Ring（Edwards Lifesciences (Shanghai) Medical Products Co., Ltd.） /(sizes T26–36).

The control device is the device that has been approved for marketing by the National Medical Products Administration, and the usage method can be found in the product instruction manual.

Instructions for use of Annuloplasty Ring used in the trial:

7.1.4.1 Annular Measurement and Selection of theAnnuloplasty Ring

Measuring the Tricuspid Valve Size:

The size of the tricuspid valve annulus is measured using a sizing device. The selection of the Annuloplasty Ring is primarily based on the dimensions of the septal leaflet attachment site. The sizing device has two notches on its straight segment, which are used to measure the annular dimensions. If the septal leaflet’s contour is not clearly visible, the sizing device can also be used to measure the anterior leaflet and its surface area. These measurements help determine the appropriate size of the Annuloplasty Ring. To ensure accurate measurements, a nerve hook can be used to gently pull on the chordae tendineae attached to the anterior papillary muscle.This maneuver helps stretch and flatten the valve leaflets, making it easier to measure the annulus accurately.

7.1.4.2 Using the Ring Holder and Holder Handle

Recommendation for Use: During the implantation of the Annuloplasty Ring, it is recommended to use the ring holder and holder handle to assist with the procedure. The holder handle is packaged separately in a non-sterile condition and must be

cleaned and sterilized before use. To use the holder handle, connect the handle’s latch to the ring holder. The holder handle features a bendable nitinol rod, which allows the handle to be adjusted to different angles to meet the needs of various surgical approaches. The nitinol rod has a shape memory property, meaning it will return to its original shape after being bent and sterilized using high-temperature steam. This feature allows the handle to be reused multiple times.

Separating the Ring Holder and Handle:

To detach the ring holder from the handle:

Hold the ring holder with one hand.

Pull the holder handle backward with the other hand.

The ring holder is for single use only and must be disposed of as medical waste after the procedure.

The holder handle can be cleaned, sterilized, and reused.

Inspection and Maintenance:

Regularly inspect the holder handle for any signs of damage, such as dullness, cracks, or wear.If any damage is found, immediately replace the handle.For replacement, contact the local representative of PerMed.

7.1.4.3 Implantation of theAnnuloplasty Ring

The Annuloplasty Ring is implanted by placing interrupted horizontal mattress sutures through the fibrous annulus. Avoid passing sutures through the His bundle area to prevent damage to the heart's electrical conduction system. To ensure proper exposure of the ring and avoid interference with the chordae tendineae, follow these steps: Gently pull the valve leaflets in a direction perpendicular to the atrial wall. Pass the suture needle from the ventricular side through the annulus, then return it toward the atrial side. Regardless of the specific technique used, the following principles must always be followed:

7.1.4.3.1 Ensure that each valve leaflet is accurately aligned with the corresponding segment of the Annuloplasty Ring.

7.1.4.3.2 Sutures should pass through the ring 2 mm away from the leaflet attachment site to preserve the natural function of the leaflets.

7.1.4.3.3 The first suture should be placed at the center of the septal leaflet to ensure proper positioning and stability of the ring.

7.1.4.4 Suturing theAnnuloplasty Ring

The Annuloplasty Ring features a suture border designed to facilitate easy passage of sutures. As shown in Figure 5, if resistance is encountered while passing the needle through the ring, withdraw the needle and reinsert it through the suture border. A green circular marking on the ring indicates the location of the suture border, aiding in proper suture placement. Sutures should be placed between the two commissures and aligned with the corresponding segments of the Annuloplasty Ring. The remaining part of the annulus should be positioned to match the size and shape of the ring, ensuring a proper fit.

7.1.4.5 Removing the Ring Holder

The ring holder features a central fixation wire at its core. To remove the holder, simply cut this fixation wire. When the holder is removed, all fixation wires are also taken out with it. After removal, the ring holder must be discarded as medical waste. Do not cut any other fixation wires besides the central one, as cutting additional wires could cause them to detach and enter the heart chamber, potentially leading to thrombus formation.

7.1.4.6 Evaluating the Repair Outcome

During tricuspid valve repair, intraoperative echocardiography is utilized to assess the function of the valve and the quality of the repair. To achieve optimal results, the following steps are critical: accurate measurement of the valve orifice size, proper selection of the Annuloplasty Ring, and optimal implantation technique. If damage to the structures below the valve is detected, additional surgical interventions may be required. After carefully measuring the size, the Annuloplasty Ring is implanted, if direct visual inspection and/or intraoperative testing still indicate that the valve regurgitation is not fully corrected, the surgeon must be prepared to remove the Annuloplasty Ring and perform artificial heart valve replacement during the same surgery.

7.1.5 Participant Selection Criteria

7.1.5.1 Inclusion Criteria

To be eligible for the trial, a patient must meet all of the following criteria:

7.1.5.1.1 Age: Between 18 and 75 years old.

7.1.5.1.2 Diagnosis: Diagnosed with tricuspid valve disease (e.g., degenerative or functional valve pathology) requiring surgical repair.

7.1.5.1.3 Heart Function: Preoperative New York Heart Association (NYHA) functional class III or lower.

7.1.5.1.4 Consent: Willing and able to provide written informed consent, comply with follow-up visits, and complete required postoperative evaluations.

7.1.5.2Exclusion Criteria

A patient will not be eligible if they meet any of the following conditions:

7.1.5.2.1 Contraindications for Annuloplasty Ring implantation or Previous tricuspid valve surgery.

7.1.5.2.2 Severe Heart Conditions:NYHA class IV heart failure,Right heart failure.

7.1.5.2.3 Researchers determine patients with increased surgical risks due to thrombus in the right atrium or right ventricle.

7.1.5.2.4 History of heart transplant.

7.1.5.2.5 Emergency surgery requirement.

7.1.5.2.6 Life expectancy less than 1 year.

7.1.5.2.7 Recent cardiac surgery, interventional procedure, or stroke within the past 3 months.

7.1.5.2.8 Kidney failure requiring dialysis.

7.1.5.2.9 Severe respiratory disease.

7.1.5.2.10 Active infective endocarditis.

7.1.5.2.11 Active systemic infection, sepsis, or unexplained fever.

7.1.5.2.12 Active gastrointestinal bleeding , peptic ulcers.or hypercoagulability in the blood.

7.1.5.2.13 Contraindications to anticoagulants/antiplatelet therapy.

7.1.5.2.14 Allergies: Known hypersensitivity to nitinol, silicone, or polyester materials.

7.1.5.2.15 Pregnancy: Currently pregnant or breastfeeding.

7.1.5.2.16 Other Trials: Participation in another drug or device study.

7.1.5.2.17 Miscellaneous: Any condition deemed unsuitable for enrollment by the investigator.

7.1.5.3 Criteria and Procedures for Stopping the Trial or Trial Treatment

7.1.5.3.1 Serious Safety Concerns

If significant safety risks are identified during the trial, immediate action will be taken to protect participants. The trial may be paused or stopped if necessary.

7.1.5.3.2 Major Errors or Deviations in the Trial Protocol

If serious mistakes or deviations in the trial plan occur, making it impossible to reliably evaluate the safety or effectiveness of the product, the trial will be discontinued.

7.1.5.3.3 Sponsor's Decision to Stop the Trial

The sponsor (the organization managing the trial) may stop the trial for reasons such as funding issues, administrative challenges, or other logistical concerns.

7.1.5.3.4 Regulatory Authority Decision

The trial will be stopped if a regulatory agency withdraws approval or orders termination.In any of these cases, the sponsor must immediately inform all participating hospitals, investigators, and ethics committees. For participants who have already received the trial treatment, the sponsor will continue to follow legal requirements and uphold responsibilities outlined in the informed consent form. Participants have the right to withdraw from the trial at any time for any reason. Researchers also have the right to withdraw participants from the trial in the event of complications, adverse events, treatment failure under standard therapy, violation of the protocol, cure, or other reasons.

Excessive participant withdrawals can compromise the reliability of the trial results. Therefore, every effort should be made to avoid unnecessary withdrawals. If a participant decides to withdraw, the investigator should make every effort to persuade them to stay and document the reasons for withdrawal in detail.Documenting Withdrawal Reasons.If a participant withdraws due to an adverse event or abnormal lab results, the investigator must record the event or abnormality in the electronic Case Report Form (eCRF). The investigator should also attempt to contact the participant (or their family) via phone, in-person visits, or other means to gather detailed information about the withdrawal.Specific Reasons for Discontinuation by the Investigator.

The investigator may also decide to discontinue a participant's involvement in the trial under the following circumstances:

Pregnancy: If the participant becomes pregnant during the trial.

Protocol Violations: If the participant fails to follow the trial procedures or requirements.

Complications or Adverse Events (AEs): If the participant experiences serious side

effects, abnormal lab results, or other medical conditions that could worsen by continuing the trial.

Non-Compliance: If the participant fails to adhere to the trial treatment or procedures.

Adverse Events or Medical Conditions: If continuing the trial poses a risk to the participant’s health.

Disease Progression: If the participant’s condition worsens, requiring discontinuation of the trial treatment.

Loss to Follow-Up: If the participant misses three scheduled follow-up visits and cannot be contacted despite repeated attempts, they will be considered lost to follow-up and withdrawn from the trial.

Severe non-compliance with study intervention;

Clinical adverse events (AE), laboratory test abnormalities or other disease conditions that would be detrimental to the subject if continued participation in the trial;

Disease progression that requires discontinuation of trial intervention;

Non-Interview: If a participant is unable to be interviewed during the specified visit period, the researcher or authorized investigator will make every effort to contact the participant before confirming their non-interview status; if a participant is missing three scheduled follow-ups and the researcher cannot reach them, they will be considered non-interviewed and will be deemed to have withdrawn from the trial, with the reason for withdrawal being non-interview.

7.1.5.4 Enrollment Time

The clinical trial is planned to be conducted at no more than 20 trial sites nationwide, with an expected enrollment period of approximately 12 months.

7.1.5.5 Expected Overall Duration of the Clinical Trial and Its Rationale

The planned duration of the clinical trial is 84 months.

Ethical approval is expected to take around 6 months. After obtaining ethical approval, completing provincial drug regulatory agency filing, and meeting other relevant policy and regulatory requirements, the trial can commence. It is planned to enroll 164 subjects within approximately 12 months after the trial starts. Clinical follow-ups will be conducted at discharge or 30 days, 3 months, 6 months, and 12 months postoperatively, recording transthoracic echocardiography (TTE), adverse events, etc. Data processing, statistical analysis, and medical summarization will be

performed after completing the 6-month follow-up, followed by registration application. A phase summary will be conducted at the 12-month postoperative mark, while long-term follow-ups and summaries will continue for up to 5 years postoperatively. Therefore, the expected overall duration of the trial is approximately 84 months.

7.1.5.6 Expected Duration of Participation for Each Subject

Each subject is expected to participate in the trial for a duration of 5 years, including preoperative screening, intraoperative heart valve annuloplasty treatment, and follow-ups at discharge or 30 days (±7 days), 3 months (±15 days), 6 months (±30 days), 12 months (±30 days), and annually from 2 to 5 years (±30 days) postoperatively.

7.1.5.7 Required Number of Subjects for the Clinical Trial

Based on statistical calculations, a minimum of 164 subjects will be enrolled.

7.1.6 Efficacy Evaluation Methods

7.1.6.1 Description of Efficacy Parameters

Primary efficacy endpoint: The success rate of valve annuloplasty repair at 6 months postoperatively, defined as no severe regurgitation and an increase in diameter ≤15% (based on a comprehensive assessment of regurgitation severity and diameter increase).

Other efficacy endpoints:

7.1.6.1.1 At discharge or 30 days postoperatively (whichever comes first), 3 months, 6 months, and 12 months:

- Degree of valve regurgitation assessed by echocardiography (evaluated according to the tricuspid regurgitation assessment index table).
- Changes in echocardiographic parameters (right atrial and ventricular size, diameter, width of the narrowest regurgitation jet, and reverse flow area).
- NYHA functional class.
- Freedom from reoperation due to tricuspid valve insufficiency.

7.1.6.1.2 Evaluation of the trial device's performance, including needle insertion smoothness and user-friendliness of the ring holder.

7.1.6.1.3 Technical success rate of the surgery (before leaving the operating room), defined as no intraoperative death, successful implantation of the annuloplasty ring, successful removal of the ring holder, and no emergency surgery or secondary

intervention related to the trial device.

7.1.6.2 Methods and Timing for Evaluating, Recording, and Analyzing Efficacy Parameters

7.1.6.2.1 Valve annuloplasty repair success is defined as no severe regurgitation and an increase in diameter ≤15% (based on a comprehensive assessment of regurgitation severity and diameter increase).

Tricuspid Regurgitation Assessment Index Table[38]

Parameter	None	Trace	Mild	Moderate	Severe
Regurgitation Jet	No regurgitation jet in the right atrium	Regurgitation jet extends ≤1 cm from the valve orifice			
Absolute Regurgitation Area			< 5cm 2	5 cm 2≤ area < 10cm 2	≥10 cm 2
Regurgitation Area Fraction			<20%	20%~33%	≥33%

Absolute regurgitation area: Measured by TTE or TEE in the standard view with the most regurgitation.

Regurgitation area fraction: Measured by TTE in the apical four-chamber view or TEE in the mid-esophageal four-chamber view, or in the standard view with the most regurgitation.

7.1.6.2.2 Diameter measurement method[39]:

Maintain the postoperative annular shape, measure the average of three diastolic annular diameters using TTE or TEE.

- Annular long diameter (A2): Measured in the standard apical four-chamber view, representing the left-right diameter of the tricuspid annulus.
- Annular short diameter (B2): Measured in the standard right ventricular inflow view, representing the superior-inferior diameter of the tricuspid annulus.

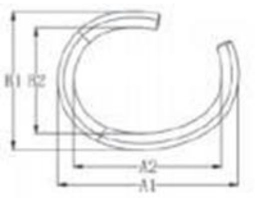

An increase in diameter is defined as the difference between the 180-day postoperative annular diameter and the 3-month follow-up diameter, divided by the 3-month follow-up diameter. An increase of more than 15% in both long and short diameters is considered significant.

7.1.6.2.3 Echocardiographic parameters: Measure right atrial and ventricular size, width of the narrowest regurgitation jet, and reverse flow area.

TTE evaluations will be conducted at discharge or 30 days (±7 days), 3 months (±15 days), 6 months (±30 days), and 12 months (±30 days) postoperatively.

7.1.6.2.4 NYHA Functional Class

Class	Description
I	Patients with cardiac disease but without limitations in physical activity. Ordinary physical activity does not cause undue fatigue, palpitation, dyspnea, or angina.
II	Patients with cardiac disease resulting in slight limitation of physical activity. Comfortable at rest, but ordinary physical activity results in fatigue, palpitation, dyspnea, or angina.
III	Patients with cardiac disease resulting in marked limitation of physical activity. Comfortable at rest, but less than ordinary activity causes fatigue, palpitation, dyspnea, or angina.
IV	Patients with cardiac disease resulting in inability to carry on any physical activity without discomfort. Symptoms of heart failure are present even at rest, and discomfort increases with any physical activity.

7.1.6.2.5 Freedom from reoperation due to tricuspid valve insufficiency.

7.1.6.2.6 Technical success rate of the surgery (before leaving the operating room):

Defined as no intraoperative death, successful implantation of the annuloplasty ring, successful removal of the ring holder, and no emergency surgery or secondary intervention related to the trial device.

7.1.7 Safety Evaluation Methods

7.1.7.1 Description of Safety Parameters

Safety endpoints:

7.1.7.1.1 At discharge or 30 days postoperatively (whichever comes first), 3 months, 6 months, and 12 months:

- Incidence of serious adverse events, including cardiovascular death, major

bleeding, severe damage to cardiac or other body structures, infective endocarditis, and thromboembolic events related to the trial device.

- All-cause mortality.
- Cardiovascular death related to the trial device.
- Major bleeding related to the trial device.
- Severe damage to cardiac or other body structures related to the trial device.
- Infective endocarditis related to the trial device.
- Thromboembolic events related to the trial device.

7.1.7.1.2 Device defects.

7.1.7.1.3 Other adverse events.

7.1.7.2 Methods and Timing for Evaluating, Recording, and Analyzing Safety Parameters

7.1.7.2.1 Serious adverse events related to the trial device are defined as events determined by the independent clinical event committee to be related, definitely related, or possibly related to the trial device, including:

- All-cause mortality: Cardiovascular and non-cardiovascular deaths from any cause.
- Cardiovascular death related to the trial device[41]]: Defined according to the Valve Academic Research Consortium (VARC) as death due to cardiac causes (e.g., myocardial infarction, cardiac tamponade, worsening heart failure), non-coronary vascular disease, surgery-related deaths (including deaths related to surgical complications or their treatment), sudden or unexplained deaths.
- Major bleeding related to the trial device[42]: Defined according to the Bleeding Academic Research Consortium (BARC) as type 3b or higher bleeding (excluding type 4).

Type	Explain
0	NH

1		Non-active bleeding that does not result in a visit to a physician; may include bleeding that results in a patient discontinuing a medication without medical advice
2		Any significant active bleeding symptoms (e.g., bleeding greater than the expected amount, including bleeding found only on imaging), excluding bleeding that fits type 3, 4, or 5, and meeting one of the following criteria: 1) bleeding requiring non-surgical medical intervention by a healthcare provider, 2) bleeding resulting in hospitalization or high-level care, or 3) bleeding requiring urgent evaluation
3	3a	There are obvious bleeding symptoms and HGB decreased by 3- 5g/dL (the correlation between HGB decrease and bleeding needs to be determined). There is obvious bleeding that requires blood transfusion
	3b	Significant bleeding symptoms and a decrease in HGB of at least 5g/dL (the correlation between the decrease in HGB and bleeding needs to be determined); cardiac tamponade; bleeding requiring surgical hemostasis (excluding bleeding from gums/nasal cavity/skin/hemorrhoids); bleeding requiring the use of vasoactive drugs
	3c	Intracranial hemorrhage (excluding microbleeds or hemorrhagic transformation; but including intradural hemorrhage); subtypes of bleeding determined by autopsy or imaging or lumbar puncture; blindness caused by intraocular hemorrhage
4 CABG-related bleeding		Intraoperative intracranial hemorrhage occurring within 48 hours; bleeding requiring re-thoracotomy for hemostasis, transfusion of ≥ 5U of packed red blood cells or whole blood within 48 hours; thoracic drainage> 2L within 24 hours
5 Fatal bleeding	5a	Possible fatal bleeding; non-pathological or imaging basis but clinically suspicious
	5b	Determine the presence of fatal bleeding with obvious signs of bleeding or with pathological or imaging evidence

- Severe damage to cardiac or other body structures related to the trial device.
- Infective endocarditis related to the trial device.
- Thromboembolic events related to the trial device[43]: Defined as myocardial

infarction, cerebral infarction, or organ embolism caused by thrombosis, with clinical or imaging evidence of arterial occlusion.

7.1.7.2.2 Surgical details and intraoperative complications will be recorded in the electronic case report form (eCRF). Safety evaluations will be conducted at discharge/30 days, 3 months, 6 months, 12 months, and annually from 2 to 5 years postoperatively, recording all adverse events. All eCRF data will be submitted to the statistical center for clinical data analysis and evaluation.

7.1.8 Trial Endpoints

Primary endpoint: The success rate of valve annuloplasty repair at 6 months postoperatively, defined as no severe regurgitation and an increase in diameter ≤15% (based on a comprehensive assessment of regurgitation severity and diameter increase).

Secondary endpoints:

7.1.8.1 At discharge or 30 days postoperatively (whichever comes first), 3 months, 6 months, and 12 months:

7.1.8.1.1 Degree of valve regurgitation assessed by echocardiography (evaluated according to the tricuspid regurgitation assessment index table).

7.1.8.1.2 Changes in echocardiographic parameters (right atrial and ventricular size, diameter, width of the narrowest regurgitation jet, and reverse flow area).

7.1.8.1.3 NYHA functional class.

7.1.8.1.4 Freedom from reoperation due to tricuspid valve insufficiency.

7.1.8.2 Evaluation of the trial device's performance, including needle insertion smoothness and user-friendliness of the ring holder.

7.1.8.3 Technical success rate of the surgery (before leaving the operating room), defined as no intraoperative death, successful implantation of the annuloplasty ring, successful removal of the ring holder, and no emergency surgery or secondary intervention related to the trial device.

7.1.8.4 At discharge or 30 days postoperatively (whichever comes first), 3 months, 6 months, and 12 months:

7.1.8.4.1 Incidence of serious adverse events, including cardiovascular death, major bleeding, severe damage to cardiac or other body structures, infective endocarditis, and thromboembolic events related to the trial device.

7.1.8.4.2 All-cause mortality.

7.1.8.4.3 Cardiovascular death related to the trial device.

7.1.8.4.4 Major bleeding related to the trial device.

7.1.8.4.5 Severe damage to cardiac or other body structures related to the trial device.

7.1.8.4.6 Infective endocarditis related to the trial device.

7.1.8.4.7 Thromboembolic events related to the trial device.

7.1.8.5 Device defects.

7.1.8.6 Other adverse events.

7.2 Trial Procedures

7.2.1 Screening (within 30 days preoperatively)

7.2.1.1 Patients must sign the informed consent form before screening.

7.2.1.2 Collect demographic data (gender, date of birth, weight, and height).

7.2.1.3 NYHA functional class, vital signs (blood pressure, pulse).

7.2.1.4 Laboratory tests: Complete blood count, blood biochemistry, coagulation function, and pregnancy test if necessary.

7.2.1.5 Electrocardiogram (ECG), echocardiography (TTE or TEE).

7.2.1.6 Record antiplatelet or anticoagulant medications.

7.2.1.7 Randomization using a central randomization system.

7.2.2 Surgical Treatment (Day 0, Surgery Day)

7.2.2.1 Monitor vital signs (blood pressure, pulse).

7.2.2.2 Perform surgery using the designated device according to randomization results, recording anesthesia method, device model, and device performance evaluation.

7.2.2.3 Transesophageal echocardiography (TEE) or transthoracic echocardiography (TTE) evaluation as needed.

7.2.2.4 Record intraoperative complications and adverse events.

7.2.2.5 Record intraoperative antiplatelet or anticoagulant medications.

7.2.3 Clinical Evaluation at Discharge or 30 Days (±7 Days) Postoperatively (Whichever Comes First)

7.2.3.1 Monitor vital signs (blood pressure, pulse).

7.2.3.2 NYHA functional class.

7.2.3.3 ECG, echocardiography (TTE).

7.2.3.4 Laboratory tests: Complete blood count, blood biochemistry.

7.2.3.5 Record adverse events.

7.2.3.6 Record antiplatelet or anticoagulant medications.

7.2.4 Clinical Evaluation at 3 Months (±15 Days) Postoperatively

7.2.4.1 NYHA functional class.

7.2.4.2 ECG, echocardiography (TTE).

7.2.4.3 Record adverse events.

7.2.4.4 Record antiplatelet or anticoagulant medications.

7.2.5 Clinical Evaluation at 6 Months (±30 Days) Postoperatively

7.2.5.1 NYHA functional class.

7.2.5.2 ECG, echocardiography (TTE).

7.2.5.3 Record adverse events.

7.2.5.4 Record antiplatelet or anticoagulant medications.

7.2.6 Clinical Evaluation at 12 Months (±30 Days) Postoperatively

7.2.6.1 NYHA functional class.

7.2.6.2 ECG, echocardiography (TTE).

7.2.6.3 Record adverse events.

7.2.6.4 Record antiplatelet or anticoagulant medications.

7.2.7 Clinical Evaluation at 2, 3, 4, and 5 Years (±30 Days) Postoperatively

7.2.7.1 Telephone follow-up.

7.2.7.2 Record adverse events.

7.2.8 Trial Study Flowchart

Experimental flow	Screening period (Preoperative)	Follow-up period (postoperative)

Visit Number		V1	V2	V3	V4	V5	V6	V7	V8	V9	V10
Days		-30d～0	Device implant a tion	Discharg e or 30d±7d	3M±15 d	6 M±30d	P12M±30 d	2Y±3 d	3Y ±30d	4Y±3 0d	5Y±3 0d
Informed Consent		X									
Inclusion/Exclusion Criteria		X									
Medical history		X									
Vital Signs (BP, P)		X	X	X							
NYHA Functional Class		X		X	X	X	X				
ECG		X		X	X	X	X				
Labo ratory Tests 1, 3	Complete Blood Count	X		X							
	Blood Biochemistr y	X									
Transesophageal Doppler echocardiograp hy (TTE) 2, 7		X 2	X 2	X	X	X	X				
Transesophageal Doppler echocardiograph y (TEE) 2, 7		X 2									
Blood or Urine Pregnancy Test (if needed)5		X									
Telephone visits								X	X	X	X
Adverse Event Recording			X	X	X	X	X	X	X	X	X
Concomitant Medications		X	X	X	X	X	X				
eCRF Completion		X	X	X	X	X	X	X	X	X	X

Notes:

1. Laboratory test results and electrocardiogram (ECG) examinations conducted within 15 days prior to signing the informed consent form are valid as baseline assessments.

2. Ultrasound examination (either Transthoracic Echocardiography [TTE] or Transesophageal Echocardiography [TEE], choose one) as a baseline assessment should be performed by an ultrasound physician at our hospital before the surgery. Intraoperative Transesophageal Echocardiography (TEE) is recommended.

3. Laboratory tests include complete blood count (white blood cells, red blood cells, hemoglobin, platelets), blood biochemistry (creatinine, alanine aminotransferase, aspartate aminotransferase, total serum bilirubin), and coagulation function (activated partial thromboplastin time, prothrombin time, thrombin time).

4. Visit 3---Before discharge or 30±7 days post-operation, whichever comes first.

5. Pregnancy test: If the subject is a woman of childbearing age, a blood or urine pregnancy test should be performed as needed.

6. Record the use of antiplatelet or anticoagulant medications.

7. Cardiac ultrasound examinations should be performed by a qualified physician at our hospital; the same subject's ultrasound examinations should be conducted at the same center; follow-up results will be independently interpreted by a senior ultrasound physician.

8. The surgery should be performed by a designated surgeon.

7.2.9 Device Usage Specifications

The sponsor will provide the "Instructions for Use" and the investigator's brochure for reference. Training will be conducted during the trial preparation phase to ensure all trial personnel are proficient in using the trial device.

7.3 Monitoring Plan

7.3.1 Clinical Trial Preparation: Before subject enrollment, the monitor will assist the investigator in submitting materials to the ethics committee, obtaining approval, signing the trial agreement, and completing provincial drug regulatory agency filing.

7.3.2 Initiation Training: All personnel involved in the trial will receive project training to ensure familiarity with GCP and protocol requirements, device operation, and clear division of responsibilities.

7.3.3 Monitoring Visits: The monitor will verify the informed consent process, inclusion/exclusion criteria, and protocol compliance. On-site monitoring visits will include:

7.3.3.1 Ensuring the informed consent process is standardized.

7.3.3.2 Verifying compliance with inclusion/exclusion criteria.

7.3.3.3 Checking for protocol deviations and ensuring deviations are recorded and reported.

7.3.3.4 Ensuring original records are timely, complete, accurate, and truthful.

7.3.3.5 Verifying timely and complete eCRF completion and ensuring data accuracy.

7.3.3.6 Ensuring trial product distribution, usage, and recovery records are complete and accurate.

7.3.3.7 Recording adverse events and ensuring serious adverse events are reported as required.

7.3.3.8 Reporting device defects as required.

7.3.3.9 Ensuring subject rights are protected as per the informed consent form.

If issues are found, they will be recorded, and the investigator will be warned and provided with targeted training. The monitor will assist the trial site in rectifying issues. A monitoring visit report will be submitted to the sponsor and the principal investigator after each visit.

7.3.4 Regular Quality Control: Quality control personnel will periodically review the work of each center, generate quality control reports, and follow up until issues are resolved. Monitors will regularly coordinate with the quality control of clinical trial institutions and the audits conducted by the sponsor, and address any identified issues through corrective actions.

8. Statistical Considerations

8.1 Statistical Design, Methods, and Analytical Procedures

8.1.1 Statistical Software and General Requirements

Software: SAS software (version 9.4 or above) will be used for analysis.
Descriptive Statistics: For continuous variables, descriptive statistics will include the number of cases, mean, standard deviation, quartiles, minimum, and maximum values. For categorical variables, descriptive statistics will include frequency and percentage.
Detailed Statistical Methods: A comprehensive statistical analysis plan (SAP) will be developed and provided separately.

8.1.2 Case Entry Analysis

The total number of cases, as well as the number of cases selected and completed at each center, will be listed to determine three analytical data sets: Full Analysis Set (FAS), Per Protocol Set (PPS), and Safety Set (SS). A detailed classification of these data sets will be provided in the SAP.

8.1.3 Demographic Data and Baseline Analysis

Descriptive statistics for demographic data and other baseline characteristics will be presented as follows:
For continuous variables: number of cases, mean, standard deviation, quartiles, minimum, and maximum values.

For count and grade data: frequency and composition ratio.
Inferences (P values) will be listed as descriptive results.

8.1.4 Analysis of the Primary Endpoint

8.1.5 Analysis of the Primary Efficacy Endpoints

The success rate of valve repair with a prosthetic ring at 6 months post-surgery will be compared using the Newcombe method or Fisher's exact test to evaluate differences between groups. The Newcombe method will be used to calculate the two-sided 95% confidence interval (CI) for the rate difference between the experimental group and the control group. Non-inferiority will be assessed based on a non-inferiority boundary value of -10%. Both PPS and FAS analyses will be conducted.

8.1.6 Analysis of Secondary Endpoints

8.1.6.1 Analysis of Secondary Efficacy Endpoints

8.1.6.1.1 Echocardiographic assessment of valve regurgitation severity at discharge or 30 days, 3 months, 6 months, and 12 months post-surgery will be compared between groups using the Cochran-Mantel-Haenszel (CMH) test.
8.1.6.1.2 Changes in echocardiographic parameters (right atrial size, right ventricular size, radial value, width of the narrowest part of the regurgitant beam, and area of reverse blood flow) at discharge or 30 days, 3 months, 6 months, and 12 months post-surgery will be compared using the t-test.
8.1.6.1.3 NYHA cardiac function classification at discharge or 30 days, 3 months, 6 months, and 12 months post-surgery will be compared between groups using the CMH chi-square test or rank-sum test.
8.1.6.1.4 The risk of reoperative tricuspid valve surgery, performance of the test instrument, and success rate of the surgical technique due to tricuspid valve insufficiency will be compared using the chi-square test or Fisher's exact test.

8.1.6.2 Secondary Safety Endpoint Analysis

8.1.6.2.1 The incidence of device-related serious adverse events (SAEs) at discharge or 30 days, 3 months, 6 months, and 12 months post-surgery will be compared between groups using the chi-square test or Fisher's exact test.

8.1.6.2.2 The incidence of all-cause mortality at discharge or 30 days, 3 months, 6 months, and 12 months post-surgery will be compared using the chi-square test or Fisher's exact test.

8.1.6.2.3 The incidence of cardiovascular death related to the test device at discharge or 30 days, 3 months, 6 months, and 12 months post-surgery will be compared using the chi-square test or Fisher's exact test.

8.1.6.2.4 The incidence of major bleeding related to the test device at discharge or 30 days, 3 months, 6 months, and 12 months post-surgery will be compared using the chi-square test or Fisher's exact test.

8.1.6.2.5 The incidence of severe damage to cardiac or other body structures related to the test device at discharge or 30 days, 3 months, 6 months, and 12 months post-surgery will be compared using the chi-square test or Fisher's exact test.

8.1.6.2.6 The incidence of device-related infective endocarditis at discharge or 30 days, 3 months, 6 months, and 12 months post-surgery will be compared using the chi-square test or Fisher's exact test.

8.1.6.2.7 The incidence of thromboembolic events related to the test device at discharge or 30 days, 3 months, 6 months, and 12 months post-surgery will be compared using the chi-square test or Fisher's exact test.

8.1.6.2.8 The incidence of adverse events from postoperative to 5-year follow-up will be compared using the chi-square test or Fisher's exact test.

8.1.6.3 Analysis of Other Safety Endpoints

Adverse Events (TEAEs): TEAEs during treatment are defined as adverse events that occur or worsen after the implantation of the investigational device. The number and incidence of the following TEAEs will be summarized by severity, System Organ Class (SOC), and Preferred Term (PT):

Adverse events

Device-related adverse events

Surgery-related adverse events

Adverse events leading to withdrawal

Serious adverse events

Summary of Adverse Events: Adverse events will be summarized according to SOC and PT, and the number of cases, frequency, and incidence will be calculated. The types of adverse events will be categorized by severity, SOC, and PT, and their

correlation with the test device will be assessed. The number of cases, frequency, and incidence will be calculated accordingly.

Laboratory Test Indicators: A cross-tabulation of laboratory test indicators, electrocardiogram (ECG), and physical examination results before and after treatment will be provided.

Abnormal Cases: Abnormal cases in laboratory indicators, ECG, physical examination, and their clinical interpretations will be listed.

Vital Signs: Descriptive statistics of vital signs after device implantation will be compared with baseline change values and actual measured values.

8.2 Calculation of sample size

8.2.1 Total sample size

According to literature reports and clinical experience, it is estimated that the success rate of valve repair with a prosthetic ring in the control group 6 months post-surgery is 95%, with a non-inferiority margin of-10%. Using a one-sided test, α=0.025, β=0.2 (power 80%), the trial group and control group are to be matched at a ratio of 1:1. Using PASS 14.0 software, it is estimated that each group will require 75 cases. Considering the risk of dislodgement during the study process, it is planned to enroll 82 cases in each group, totaling 164 cases.

8.2.2 Number of clinical trial cases for each disease and the reasons for their determination

According to the design requirements of this trial protocol,patients with tricuspid valve insufficiency were selected, and the number of samples was determined according to the statistical design to be 164.

8.2.3 Minimum and maximum number of subjects in each clinical trial institution and reasons for multicenter clinical trials

This trial is expected to enroll 164 participants and will be conducted simultaneously at multiple clinical trial sites, implementing competitive enrollment. In principle, the number of participants enrolled at each center will be evenly distributed as much as possible to ensure adequate center representation. However, considering feasibility and enrollment progress, a competitive enrollment approach is adopted to maintain overall balance between groups.

8.3 Significance level and confidence level of clinical trials

Non-inferiority testing is represented by the two-sided 95% confidence interval of the difference between groups. All statistical tests are conducted as two-sided tests; a P-value less than or equal to 0.05 will be considered statistically significant. With 164 patients selected, there is over 80% confidence that the efficacy of the treatment group is non-inferior to the control group.

8.4 Expected fall-off rate

The expected fall rate is about 10%.

8.5 Criteria for qualified/unqualified clinical trial results

Determining whether the experimental results are qualified from a statistical perspective is equivalent to verifying the initial hypothesis test. For this study, it is necessary to meet the non-inferiority test for the primary efficacy endpoint, which involves calculating the rate difference between the treatment group and the control group using the Newcombe method for the success rate of valve repair with prosthetic rings at 6 months post-surgery, and assessing whether the efficacy of the treatment group is not inferior to that of the control group based on a non-inferiority margin of-10%.

8.6 Criteria and reasons for terminating the trial on statistical grounds

A non-inferiority design is adopted, with the non-inferiority margin δ set at -10%.

H0 (null hypothesis): $P1 - P2 \leq \delta$

H1 (alternative hypothesis): $P1 - P2 > \delta$

Where P1 is the success rate of valve repair with a prosthetic ring at 6 months post-surgery in the experimental group, and P2 is the success rate of valve repair with a prosthetic ring at 6 months post-surgery in the control group. δ represents the non-inferiority margin.

8.7 Statistical methods for all data, including processing of missing, unused or erroneous data (including dropout and withdrawal) and unreasonable data

When the main indicators are missing, the last observation value carry-over method (LOCF) is used to fill in the data. Other indicators are analyzed using the actual collected data.

8.8 Report deviations from the planned statistical procedures

After the final draft of the statistical plan is finalized, if any changes are needed during the statistical analysis, the sponsor,investigator and statistician should agree to make any changes. The statistical analysis report and summary report should clearly specify the additional statistical analysis results.

8.9 Selection criteria and reasons for subjects included in the analysis

Full analysis set (full analysis set, FAS): The collection of all cases randomized to the group and who received surgical implantation devices.

Conformity set (per protocol set, PPS): A subset of the full analysis set, a data set generated by subjects who are fully compliant with the protocol, compliance including treatment received, availability of measurement of primary endpoint indicators, and no significant deviation from the protocol. PPS analysis is used for primary efficacy endpoints.

Safety Dataset (safety set, SS): All actual data that were randomly enrolled and underwent surgical implantation of devices and had post-implant safety metrics recorded. SS analysis was used for primary safety metrics.

Efficacy analysis will be performed on the full analysis set and the compliant set. All baseline demographic data analysis will be performed on the full analysis set and safety evaluation will be performed on the safety set.

8.10 Exclusion of special information and its rationale in the verification of assumptions (if applicable)

Not applicable.

9. Data management

This test uses electronic data management, using DAS for EDC (V6.0). The following lists the main data management processes, and other details are shown in the Data Management Plan (DMP).

The DMP is a guiding document for data management written by the Data Administrator (DM) and approved by the sponsor. The data management work will be carried out according to the time, content and method defined in the DMP.

9.1 EDC data management

9.1.1 Electronic Case Report Form (eCRF): The data administrator constructs it according to the trial protocol design and sets up logical verification according to the data verification plan (DVP). It is released for use after testing and approval by the

sponsor.

9.1.2 Data entry: The data of eCRF comes from the original records, and the data entry personnel shall timely enter the subject visit data into EDC according to the instructions of eCRF.

9.1.3 Source Data Field Verification (SDV): The monitor shall check the consistency between eCRF data and source data, and raise questions if there are any problems.

9.1.4 Data Questions and Answers: Questions come from system questions of EDC logical verification, manual questions from monitors and data administrators, etc., and researchers need to answer questions in time. Data administrators and monitors will reply to questions, and questions can be sent again if necessary until the data is "cleaned".

9.1.5 Signature of the investigator: After data entry is completed and approved by SDV, the investigator shall sign the electronic signature for confirmation. If there is any data revision after signing, the signature shall be signed again.

9.1.6 Database Locking: After the database locking record is signed by the principal investigator, sponsor, statistical analyst and data manager, the data manager locks the database.

9.1.7 Database submission: The data administrator submits the database to the statistician.

9.1.8 eCRF Archiving: Each subjects eCRF is generated as a PDF electronic document and saved.

9.1.9 Data management report: written by data administrator.

9.1.10 EDC shutdown: After the statistical analysis is completed, the data administrator shuts down the database.

9.2 External data transfer

Sign an external data transfer agreement to manage external data according to the DMP.

9.3 Medical coding

Adverse events were coded using the MedDRA (version 23.0 or above) dictionary, and drug combinations were classified by WHO ATC.

10. Feasibility analysis

10.1 Analysis of the likelihood of success

10.1.1. In order to eliminate potential risks of the subjects, the sponsor has conducted physical, chemical and biological tests and evaluations on the investigational product to prove its safety. The investigational product has passed the registration inspection of the medical device inspection center, and its technical specifications meet the registration standards.

10.1.2. The preclinical animal study of the test product has been completed, and the preliminary results show that the test product can function normally together with the original valve structure, and its safety and effectiveness are good.

10.1.3. The sponsor has made a detailed analysis of the possible risks of the product in application when developing and testing the product. The risk analysis and evaluation are carried out in accordance with "Risk Management of Medical Devices for the Application of Medical Devices". The analysis results show that all risks are within an acceptable range and the clinical benefits outweigh the use risks.

10.1.4. This clinical trial is conducted in compliance with the Good Clinical Practice for Medical Devices and relevant regulations of the National Medical Products Administration. The protocol must be approved by the ethics committee before implementation. Participants or their guardians must sign an informed consent form to participate in the trial, ensuring participant compliance. Investigators strictly adhere to case selection criteria and follow the design of the trial protocol to minimize confounding factors, reduce risks, and increase the likelihood of successful clinical trials.

In addition, since the clinical institutions undertaking this trial all have complete equipment and technical resources, and the clinical

trial leader has senior clinical experience, it is expected that this trial can achieve the verification purpose designed in the trial.

10.2 Analysis of the possibility of failure

Factors such as failure to strictly implement clinical protocols, failure to standardize operation and testing, loss of follow-up and withdrawal may lead to the failure of this trial, but these factors arestill within the controllable range, so the possibility of failure of this trial is small.

11. Quality control of clinical trials

11.1 Training for clinical trials

Before the clinical trial is started, the relevant personnel of the clinical trial should be trained, and the investigators of each center should be trained at the launch meeting, including clinical protocol, informed process, instrument use, document filling and other aspects. During the trial, the investigators and relevant personnel of the trial should be trained according to the actual clinical situation.

11.2 Monitoring of clinical trials

In the process of clinical trial, the sponsor appointed inspectors visit each research center regularly according to the clinical supervision plan, and the sponsor designated inspectors supervise the standardization and authenticity of the clinical trial process according to the actual situation, so as to ensure that all contents of the research protocol are strictly complied with. The original data is checked to ensure that the eCRF filling is true, complete and correct.

11.3 Audit of clinical trials

According to the progress of clinical trials, including the period of enrollment and follow-up, professional auditors shall be organized for audit when necessary to ensure the accuracy, completeness and timeliness of data as well as compliance of the clinical process.

11.4 Preservation of original data

The original data of this trial include signed informed consent, records of the use of the trial product, relevant laboratory test reports, medical records and other related records, etc., which should be kept in the national medical device clinical trial institutions of each research center, and the clinical trial data shall be kept for 10 years after the completion of the clinical trial, and the sponsor enterprise shall keep the relevant clinical trial data for a long time.

12. Ethical issues and informed consent in clinical trials

12.1 Ethical considerations

This clinical trial is conducted in compliance with the requirements of the Helsinki Declaration and relevant national regulations. The investigator is responsible for providing the ethics committee with the clinical trial protocol, informed consent form and any information provided to the subjects in order to obtain independent approval

documents for the implementation of the clinical trial, which can be carried out only after the ethics committee has approved it.

During the clinical trial, any problems related to the safety of the clinical trial, such as changes in the clinical trial protocol or informed consent form of the subjects, and serious adverse events in the clinical trial must be reported to the ethics committee in a timely manner. The end or early termination of the clinical trial must also be reported to the ethics committee.

For participants in the trial, an informed consent form must be signed. For incapacitated participants, if the ethics committee agrees in principle and the researcher believes that the participants participation is in their best interest, these patients can also enter the trial, but their legal guardian or authorized representative must sign and date it before the trial begins. Participants have the right to withdraw from the clinical trial at any stage.

12.2 Approval of the test plan

The same version of the clinical trial protocol shall be submitted to each center and implemented after being approved by the ethics committee. During the clinical trial, any revision of the clinical trial protocol shall be implemented only with the written approval of the ethics committee.

12.3 Process of informed consent and text of informed consent form

12.3.1 The Informed Consent Form can only be used after it has been approved by the Ethics Committee. If the Informed Consent Form is revised during the experiment, the revised Informed Consent form shall be reviewed and approved by the Ethics Committee again before use. After the revised Informed Consent is approved and sent to the clinical trial institution for record, all subjects who have not completed the trial process must sign the newly revised Informed Consent, and all subjects who have completed the final follow-up do not need to sign the newly revised Informed Consent.

12.3.2 Before participants in the clinical trial, investigators should fully explain the detailed circumstances of the clinical trial to the participant or their family members, guardians, and legal representatives, including known and foreseeable risks and potential adverse events. After a thorough and detailed explanation, the participant or their legal representative signs their name and date on the Informed Consent Form, and the investigator who executed the informed consent must also sign their name and date

on the Informed Consent Form.

13. Regulations on the reporting of adverse events and device defects

13.1 Adverse events

Adverse event refers to adverse medical events that occur during the trial but are not necessarily related to the investigational product.

Adverse event severity determination:

Mild-does not affect the subjects daily activities;

Moderate-This type of AE causes mild discomfort or consideration of treatment measures in the subject and may affect the subjects daily activities;

Severe-This type of AE interferes with the subjects normal daily life and requires systematic medication or other treatment.

All adverse events that occurred during the trial must be truthfully recorded in the adverse event form. The investigator should give targeted treatment and follow up for adverse events until symptoms disappear or stabilize.

The relationship between adverse events and research instruments

Certain-there is clear evidence that it has a causal relationship and other possible causes have been excluded.

It is likely that the AE was known to be associated with the study treatment and there is a reasonable likelihood that the study treatment caused the AE or that there is a temporal relationship between the study treatment and the AE. A reasonable likelihood means that there is evidence suggesting a causal relationship between the study treatment and the AE.

There may be a possible association—there is some evidence suggesting a causal relationship (for example, the event occurred within a reasonable time interval after the use of the investigational device). Then, other factors can also play a role in the event (for example, the subjects clinical condition, other concomitant events). Although an AE is initially classified as "possible association" upon discovery, more information should be obtained as requested, and if appropriate, it can be upgraded to "probably associated" or "definitely associated."

May not be relevant ---- Clinical events include abnormal laboratory results

where temporal relationships with the study treatment are unlikely to support causation (e.g., the event did not occur within a reasonable time interval after the use of the test device) and where other underlying medical conditions give a reasonably plausible explanation (e.g., the subject's clinical condition, other concomitant treatments).

Irrelevant-The AE is completely independent of the study treatment,or there is evidence that the event is completely related to another cause (the physician must document the cause of the AE), or there is no reasonable likelihood that the study treatment caused the AE, there is no temporal relationship between the study treatment and the occurrence of the AE, or another cause of the AE has been identified.

13.2 Serious adverse events and device defects

Serious adverse events refer to incidents occurring during clinical trials that result in death or a significant deterioration of health, including fatal diseases or injuries, permanent defects in body structure or function, requiring hospitalization or extended hospital stays, necessitating medical or surgical intervention to prevent permanent defects in body structure or function ; leading to fetal distress, fetal death, congenital abnormalities, or congenital defects.

Instrumental defects refer to the unreasonable risks that may endanger human health and life safety in the normal use of medical devices during clinical trials, such as label errors, quality problems and faults.

13.3 Reporting procedures and contact information

In the event of serious adverse events in clinical trials, investigators should immediately take appropriate treatment measures for the subjects and report in writing to the corresponding ethics committee and the provincial, autonomous region, or municipal drug administration department and health authority where the clinical trial institution is located within 24 hours. They should also report in writing to their affiliated clinical trial institution and notify the sponsor. For fatal events, the clinical trial institution and investigators should provide all necessary information to the ethics committee and the sponsor.

Researchers should record the defects of the devices found in the clinical trial process, analyze the causes of the events together with the sponsor, form a written analysis report, and report to the clinical trial institution and the ethics committee

whether to continue, suspend or terminate the trial.

Contact information of the applicant enterprise:

Name: Sun Yufang

Tel: 180 1900 8461

E-mail: yufang.sun@cardim ed.com.cn

Address: 1st to 3rd floors, Building 7, No.9 Tianfu Street, Daxing District, Beijing

14. Deviation from and amendment of the clinical trial protocol

If there is a major deviation from this trial protocol, or a large number of subjects seriously violate the clinical protocol, and there is a clear impact on the primary endpoint of the test product, the sponsor shall negotiate with the lead unit investigator, obtain the approval of the researchers of each center, submit an amendment, and state the reasons for the amendment.

15. Direct access to source data and files

When the sponsor, clinical trial institution and investigator allow relevant monitoring, verification, ethics committee and management department inspection of the clinical trial, relevant personnel can directly access the source data/document.

16. Finance and insurance

See the clinical trial cooperation agreement signed by the sponsor and each trial unit for details.

17. Contents that should be covered in clinical trial reports

According to the requirements of medical device clinical trial quality management, the clinical trial report should be consistent with the clinical trial protocol, including the following contents:

17.1 General information;

17.2 Summary;

17.3 Introduction;

17.4 Purpose of clinical trials;

17.5 Clinical trial method;

17.6 Clinical trial content;

17.7 General clinical data;

17.8 Test medical devices and control medical devices or control diagnostic methods;

17.9 Statistical analysis and evaluation methods used;

17.10 Clinical evaluation criteria;

17.11 Organization structure of clinical trials;

17.12 Ethical situation statement;

17.13 Clinical trial results;

17.14 Adverse events found in clinical trials and their handling;

17.15 Analysis and discussion of clinical trial results, especially indications, scope of application, contraindications and precautions;

17.16 Clinical trial conclusion;

17.17 Existing problems and recommendations for improvement;

17.18 List of test personnel;

17.19 Other matters requiring clarification.

18. Confidentiality principle

Researchers have the responsibility to protect the life, health, dignity, integrity, self-determination, and privacy of research participants, ensuring the confidentiality of their personal information. Participants must be informed that their participation in the trial and their personal data during the trial are confidential. However, ethics committees, drug regulatory authorities, or sponsors may, when necessary for work purposes, review the personal data of participants according to established procedures.

19. Agreement on publication of test results

Without the written consent of the sponsor, researchers must not disclose data or any other information related to this clinical trial to third parties or for any other purpose. If a researcher publishes or otherwise publicly describes the results obtained from the investigational product of this clinical trial, prior written consent from Party

A is required. The sponsor should review and respond to the proposed disclosure within 30 working days.

Without the consent of the investigator, the sponsor will not use the name of the investigator for commercial purposes such as promotional and advertising activities for the investigational device. However, when conducting clinical trials of the investigational device and applying for production approval from the National Medical Products Administration, the sponsor may use all results obtained during the clinical trial process, including the clinical trial report of the medical device, without the investigators consent.

20.Duties of the parties

In the clinical trial, all parties responsibilities follow the requirements of medical device clinical trial quality management standards, which are briefly described as follows:

20.1 Responsibilities of the sponsor

20.1.1 The sponsor is responsible for initiating, applying for, organizing and monitoring clinical trials, and is responsible for the authenticity and reliability of clinical trials.

20.1.2 The sponsor is responsible for organizing the development and modification of the investigators manual, clinical trial protocol, informed consent form, electronic case report form, relevant standard operating procedures and other related documents, and is responsible for organizing the necessary training for the clinical trial.

20.1.3 The sponsor shall select experimental institutions and their investigators from qualified clinical trial institutions according to the characteristics of experimental medical devices. Before signing the clinical trial agreement with the clinical trial institution, the sponsor shall provide the clinical trial institution and the investigator with the latest investigator manual and other relevant documents for them to decide whether they can undertake the clinical trial.

20.1.4 During the clinical trial, when the sponsor obtains important information affecting the clinical trial, it shall timely modify the investigators manual and relevant documents, and submit them to the medical device clinical trial management department of the clinical trial institution for review and approval by the ethics

committee.

20.1.5 The sponsor shall be responsible for the safety of the investigational medical device in the clinical trial. If it is found that it may affect the safety of the subject or the implementation of the trial may change the approval of the ethics committee for the continuation of the trial, the sponsor shall immediately notify all clinical trial institutions and investigators and make corresponding treatment.

20.1.6 If the sponsor decides to suspend or terminate a clinical trial, they must notify all medical device clinical trial management departments of the clinical trial institutions within 5 days and provide a written explanation of the reasons. The medical device clinical trial management department of the clinical trial institution should promptly inform the corresponding researchers and ethics committees. For suspended clinical trials, they shall not be resumed without the consent of the ethics committee. After the completion of the clinical trial, the sponsor must inform in writing the provincial, autonomous region, or municipal drug regulatory authority where they are located.

20.1.7 The sponsor shall ensure that all investigators conducting the clinical trial strictly adhere to the protocol. If any investigator or clinical trial institution is found not complying with relevant laws, regulations, this standard, and the protocol, it should be promptly pointed out and corrected; if the situation is serious or persists without correction, the trial should be terminated, and a report should be made to the provincial, autonomous region, or municipal drug regulatory authority where the clinical trial institution is located, as well as to the National Medical Products Administration.

20.1.8 The sponsor shall bear the cost of treatment and corresponding economic compensation for subjects who suffer injuries or deaths related to clinical trials, except for damages caused by the fault of medical institutions and their medical personnel in the diagnosis and treatment activities.

20.1.9 The sponsor shall be responsible for monitoring the clinical trial and select qualified monitors to fulfill their duties. To ensure the quality of the clinical trial, the sponsor may organize independent auditors with appropriate training and experience to verify the conduct of the trial and assess whether it meets the requirements of the protocol.

20.1.10 For serious adverse events and device defects that may lead to serious adverse events, the sponsor shall report to the registered drug regulatory authority and the same-

level health administration department within 5 working days of becoming aware, while also informing other clinical trial institutions and investigators involved in the trial. The sponsor shall promptly notify the ethics committee of the clinical trial institution through its medical device clinical trial management department.

20.2 Responsibilities of clinical trial institutions and investigators

20.2.1. Before accepting a clinical trial, the clinical trial institution shall evaluate the relevant resources and assess the qualifications of the investigators according to the characteristics of the medical device used in the trial, so as to decide whether to accept the clinical trial.

20.2.2. Clinical trial institutions shall properly keep clinical trial records and basic documents in accordance with the agreement with the sponsor.

20.2.3. Before clinical trials, the medical device clinical trial management department of the clinical trial institution shall cooperate with the sponsor to apply to the ethics committee and submit relevant documents in accordance with regulations.

20.2.4. The investigator shall ensure that the relevant staff involved in the trial are familiar with the principles, applicable scope, product performance, operation method, installation requirements and technical indicators of the medical device used in the trial, understand the preclinical research data and safety data of the medical device used in the trial, and master the prevention of risks that may arise from clinical trials and emergency treatment methods.

20.2.5. Researchers shall ensure that all participants in clinical trials fully understand the trial protocol, relevant regulations, characteristics of medical devices used in the trial, and their responsibilities related to the clinical trial. They must also ensure that a sufficient number of eligible participants meet the inclusion criteria for the trial and that there is adequate time during the trial period specified in the protocol to safely conduct and complete the clinical trial according to relevant regulations.

20.2.6. Researchers are responsible for recruiting participants and interviewing them or their guardians. Researchers must inform participants about the medical devices used in the trial and provide detailed information related to the clinical trial, explaining potential benefits and known, foreseeable risks. They must also obtain a signed and dated informed consent form from the participant or their guardian.

20.2.7. Researchers are responsible for making medical decisions related to clinical trials. In the event of adverse events associated with the clinical trial, the clinical trial institution and researchers should ensure that participants receive adequate and timely treatment and management. When participants develop complications requiring treatment or management, researchers should promptly inform them. For any identified device defects, researchers should work with the sponsor to analyze the cause of the incident, prepare a written analysis report, and submit it to the ethics committee.

20.2.8. In the event of a serious adverse event in clinical trials, the investigator shall immediately take appropriate treatment measures for the subject and report in writing to the clinical trial management department of the medical device at the affiliated clinical trial institution, and notify the sponsor in writing. The clinical trial management department shall report in writing to the corresponding ethics committee and the provincial, autonomous region, or municipal drug administration and health authorities where the clinical trial institution is located within 24 hours. For fatal events, the clinical trial institution and the investigator shall provide all necessary information to the ethics committee and the sponsor.

20.2.9. The investigator shall ensure that the clinical trial data is accurately, completely, clearly and timely recorded in the electronic case report form. The electronic case report form shall be signed by the investigators name, any modification of data shall be signed by the investigator and dated, and the original record shall be retained, which shall be clear and identifiable.

20.2.10. At the end of the clinical trial, the investigator shall ensure that all records and reports are completed. At the same time, the investigator shall also ensure that the number of trial medical devices received is consistent with the number used, discarded or returned, and that the remaining trial medical devices are properly disposed of and recorded in archives.

References

[1] Liu X, Xiao YB. Current status of surgical treatment for functional tricuspid regurgitation secondary to left heart disease [J]. Journal of Regional Anatomy and Operative Surgery, 2014, 23(03): 300-302.

[2] Zhong RH, Yuan YD, Huang JH, et al. Application value of C-shaped flexible ring in the treatment of functional tricuspid insufficiency [J]. China Journal of Pharmaceutical Economics, 2017, 12(06): 105-107.

[3] Wang ZW, Liu WY, Zhang BR, et al. Cardiac Surgery [M]. Beijing: People's Military Medical Press, 2003: 1311-1312.

[4] Liao SJ, Huang HL, Fei HW, et al. Pathophysiological changes of right heart remodeling caused by tricuspid regurgitation [J]. The Journal of Practical Medicine, 2014, 30(19): 3042-3045.

[5] Wechsler AS.Mitral valve surgery for functional mitral regurgitation:should moderate to severe tricuspid regurgitation be treated? A propensity score analysis[J].J Thorac Cardiovasc Surg,2009,137(2):267-268.

[6] Chan V,Burwash IG,Lam BK,et al. Clinical and echocardiographic impact of functional tricuspid regurgitation repair at the time of mitral valve replacement[J].Ann thorac surg,2009,88(4):1209-1215.

[7] Bonow RO,Carabello BA,Chatterjee K,et al.2008 focused update incorporatd into the ACC/AHA 2006 guidelines for the management of patients with valvular heart disease: a report of the American College of Cardiology/American Heart Association Task Force on Practice Guide-lines(Writing Committee to revise the 1998 guideline for the management of patients with valvular heart disease):endorsed by the Society of Cardiovascular Angiography and interventions and Society of Thoracic Surgeons [j]. Am Coll Cardiol,2008,52(13):e1-e142.

[8] Vahanian A, Alfieri o, Andreotti F, et al. Guidelines on the management of valvular heart disease version (2012):The Joint Task Force on the Management of Valvular Heart Disease of the European Society of Cardiology(ESC) and the European Association for Cardio-Thoracic Surgery(EACTS)[J].Eur Heart,2012,33(19)：2451-2496.

[9] Calafiore AM, Bartoloni G, Amri, et al. Functional tricuspid regurgitation and the right ventricle: What we do not know is more than we know[J].Expert review of

cardiovascular therapy,2012,10(11):1351-66.

[10] Deloche A,Guerinon J,Fabiani J N,et al.Anatomical study of rheumatic tricuspid valve diseases: Application to the study of various valvuloplasties[J].Ann Chir Thorac Cardiovasc,1973,1(2):343-9.

[11] Kobayashi J,Kawashima Y,Matsuda H,et al.Prevalence and risk factors of tricuspid regurgitation after correction of tetralogy of Fallot[J]. Thor Cardiovascular Surg,1991,10(2):611-6.

[12] Bonow RO,Carabello BA,Chatterjee K,et al.2008 Focused Update Incorporated Into the ACC/AHA 2006 Guidelines for the Management of Patients With Valvular Heart Disease[J].Circulation, 2008, 118:e523-e661.

[13] Vahanian A,Alfieri O,Andreotti F,et al. Guidelines on the management of valvular heart disease (version 2012):The Joint Task Force on the Management of Valvular Heart Disease of the European Society of Cardiology (ESC)and the European Association for Cardio-Thoracic Surgery (EACTS)[J].G Ital Cardiol(Rome),2013,14(3):167-214.

[14] Nishimura RA,Otto CM,Bonow RO,et al.2014 AHA/ACC Guideline for the management of patients with valvular heart disease:executive summary:a report of the American College of Cardiology/American Heart Association Task Force on Practice Guidelines[J].J Am Colle Cardiol,2014.6(3):2438-88.

[15] Dreyfrus G.Functional tricuspid regurgitation.A need to revise our understanding[J].J Am Coll Cardiol,2015,6(5):2331-6.

[16] Marquis-Gravel G,Bouchard D ,Perrault LP,et al.Retrospective cohort analysis of 926 tricuspid valve surgeries: Clinical and hemodynamic outcomes with propensity score analysis[J] .Am Heart ,2012,16(3):851-858el.

[17] Van de Veire NR,Braun J,Delgado V,Versteegh,et al.Tricuspid annuloplasty prevents right ventricular dilatation and progression of tricuspid regurgitation in patients with tricuspid annular dilatation undergoing mitral valve repair[J]. The Journal of Thoracic and Cardiovascular Surgery,2010,141(6).

[18] Fang Z, Meng W, Zhang EY. Short-term efficacy analysis of three tricuspid valvuloplasty techniques for tricuspid regurgitation [J]. Chinese Journal of Reparative and Reconstructive Surgery, 2013, 27(10): 1230-1233.

[19] Alessandro Parolari, Fabio Barili, Alberto Pilozzi, Davide Pacini. Ring or Suture Annuloplasty for Tricuspid Regurgitation? A Meta-Ana l ysis Review[J]. The Annals of Thoracic Surgery,2014,98(6).

[20] Sung Ho Shinn , Victor Dayan , Hartzell V. Schaff,et al.Outcomes of ring versus suture annuloplasty for tricuspid valve repair in patients undergoing mitral valve surgery[J].The Journal of Thoracic and Cardiovascular Surgery,2016, 152 (2):406-415.e3.

[21] Chen HZ, Zou LJ, Xu ZY, et al. Mid-term evaluation of tricuspid valve annuloplasty using rigid prosthetic rings [J]. Chinese Journal of Clinical Thoracic and Cardiovascular Surgery, 2005, 12(2): 116-117.

[22] Carpentier A, Deloche A, Hanania G, et al. Surgical management of acquired tricuspid valve disease[J].J Thorac Cardiovasc Surg,1974,Jan,67(1):53-65.

[23] Duran CG,Ubago JL. Clinical and hemodynamic performance of a totally flexible prosthetic ring for atrioventricular valve reconstruction[J].Ann Thorac Surg,1976,22(5):458-463.

[24] Goksin I, Yilmaz A, Baltalarli A, et al. Modified semicircular constricting annuloplasty (Sagban's annuloplasty) in severe functional tricuspid regurgitation:alternative surgical technique and its midterm results[J].J Card Surg,2006,21(2):172-175.

[25] McCarthy JF, Cosgrove DM 3rd. Tricuspid valve repair with the Cosgrove Edwards Annuloplasty System[J].Ann Thorac Surg, 1997, 64 (1):267-268.

[26] Gatti G, Pacilli P, Pugliese P. Tricuspid valve annuloplasty using a partial flexible ring: midterm follow-up[J].Ital Heart J, 2003, 4(2):121-124.

[27] McCarthy PM,Bhudia SK, Rajeswaran J, et al. Tricuspid valve repair:Durability and risk factors for failure[J]. Thorac Cardiovasc Surg, 2004,127:674-685.

[28] Filsoufi F, Salzberg SP, Coutu MA, et al. A three-dimensional ring annulopl asty for the treatment of tricuspid regurgitation[J]. Ann Thorac Surg2006,81(6):2273-2277.

[29] Fukuda S,Gillinov AM, McCarthy PM, et al. Echocardi ographic follow-up of tricuspid annuloplasty with a new three-dimensional ring in patients with functional tricuspid regurgitate on[J]. J Am Soc Echocardioger, 2007, Nov, 20(11):1236-1242.

[30] De Lazerda DJ, Cohen O, Marelli D, et al. Tricuspid valve repair using autologous pericardium annuloplasty in adults[C]..Heart Surg Forum, 2008,11(4):E4-8.

[31] Su LD, Liu HY. Surgical treatment of functional tricuspid regurgitation [J]. Chinese Journal of Evidence-Based Cardiovascular Medicine, 2017, 9(11): 1390-1393.

[32] Nie YH, Zhang SD, Zhang R, et al. Clinical efficacy of tricuspid valvuloplasty versus valve replacement for severe functional tricuspid regurgitation with right heart failure [J]. Chinese Journal of the Frontiers of Medical Science (Electronic Version), 2018, 10(7): 112-115.

[33] Wu XJ, Zhang SY, Shang ZR. Clinical efficacy of tricuspid valvuloplasty using MC3 annuloplasty ring for rheumatic valvular disease with tricuspid regurgitation [J]. Chinese Journal of Cardiovascular Research, 2019, 17(4): 356-359, 384.

[34] Zhou W, Xin J, Du W, et al. Efficacy analysis of annuloplasty rings in the treatment of functional tricuspid regurgitation [J]. Chinese Journal of Modern Operative Surgery, 2012, 16(1): 30-32.

[35] Chen TB, Liu J, Huang HL. Therapeutic effect of annuloplasty rings on functional tricuspid regurgitation [J]. South China Journal of Cardiovascular Diseases, 2019, (2): 163-167.

[36] Wu X, Wang JX, She K. Progress in tricuspid valvuloplasty for tricuspid regurgitation [J]. Chinese Journal of Cardiovascular Research, 2015, 13(10): 874-877.

[37] Zhang R, Wang X, Yang XH, et al. Observation on the therapeutic effect of prosthetic ring annuloplasty on functional tricuspid regurgitation [J]. China Medical Engineering, 2018, 26(5): 49-52.

[38] Meng H, Wang H, Pan SW, et al. Comprehensive evaluation of secondary tricuspid regurgitation by echocardiography [J]. Chinese Journal of Ultrasound in Medicine, 2015, 31(4): 326-328.

[39] Zheng S, Jiao YQ, Meng X. Imaging of mitral annulus diameter after 3D annuloplasty ring implantation [J]. Journal of Cardiovascular and Pulmonary Diseases, 2018, 37(6): 547-550.

[40] Stone G, Adams D, Abraham W, et al. Clinical Trial Design Principles and Endpoint Definitions for Transcatheter Mitral Valve Repair and Replacement: Part 2: Endpoint Definitions [J]. JACC,2015,66(3):308-321.

[41] A Pieter Kappetein, Stuart J. Head, Philippe Genereux,et al.Updated standardized endpoint defnitions for transcatheter aortic valve implantation: the Valve Academic Research Consortium-2 consensus document[J]. European Heart Journal，2012，33：

2403-2418.

[42] Mehran R, Rao S V, Bhatt D L, et al. Standardized bleeding definitions for cardiovascular clinical trials: a consensus report from the Bleeding Academic Research Consortium[J]. Circulation, 2011, 123(23):2736-47.

[43] Orgel R, Wojdyla D, Huberman D,et al.Noncentral Nervous System Systemic Embolism in Patients With Atrial Fibrillation: Results From ROCKET AF (Rivaroxaban Once Daily, Oral,Direct Factor Xa Inhibition Compared With Vitamin K Antagonism for Prevention of Stroke and Embolism Trial in Atrial Fibrillation)[J]. Circ Cardiovasc Qual Outcomes，2017，10(5):1-3.
