# Supplementary material for: Efficacy and safety of PM-AR-T versus edwards MC3 rings in tricuspid regurgitation: A non-inferiority, randomized controlled trial
Source: PLoS One. 2025 Dec 12;20(12):e0333891. doi: 10.1371/journal.pone.0333891 (PMC12700415; doi:10.1371/journal.pone.0333891)
Supplement: Attachment — Submitted filename: Rebutal letter-07.24 revised.docx [file pone.0333891.s010.docx]

**Manuscript ID:** PONE-D-25-24627

**Title:** Efficacy and Safety of PM-AR-T versus Edwards MC3 Rings in Tricuspid Regurgitation: A Non-inferiority, Randomized Controlled Trial

**Journal:** PLOS ONE

**Response to Reviewers’ comments**

Dear Kamal Sharma,

Thank you for your letter and the reviewers’ constructive feedback on our revised manuscript. We have carefully addressed all points raised in the reviewers’ comments. We have provided an itemized summary of our responses below and uploaded both clean and tracked versions of the revised manuscript, to facilitate further review.

We thank you again for your time spent on reviewing our work，and value this opportunity to improve our manuscript. We hope that you find our responses satisfactory and look forward to further feedback from you.

Best regards,

On behalf of my co-authors,

Dongjing Wang

Department of Cardiothoracic Surgery, Nanjing Drum Tower Hospital, The Affiliated Hospital of Nanjing University Medical School, Nanjing 210009, China

glwdj@nju.edu.cn

**In the response letter attachment:**

**Response to editor and referees:**

**Editor**

*When submitting your revision, we need you to address these additional requirements.*

*1. Please ensure that your manuscript meets PLOS ONE's style requirements, including those for file naming. The PLOS ONE style templates can be found at*

*https://journals.plos.org/plosone/s/file?id=wjVg/PLOSOne_formatting_sample_main_body.pdf and*

[*https://journals.plos.org/plosone/s/file?id=ba62/PLOSOne_formatting_sample_title_authors_affiliations.pdf*](https://journals.plos.org/plosone/s/file?id=ba62/PLOSOne_formatting_sample_title_authors_affiliations.pdf)

*2. Thank you for stating the following financial disclosure:“Beijing Permed Biomedical Engineering Co., Ltd.”*

*Please state what role the funders took in the study. If the funders had no role, please state: "The funders had no role in study design, data collection and analysis, decision to publish, or preparation of the manuscript."*

*If this statement is not correct you must amend it as needed. Please include this amended Role of Funder statement in your cover letter; we will change the online submission form on your behalf.*

Response: Thank you for the feedback and editorial guidance. We have reviewed the manuscript and ensured that it meets PLOS ONE’s style requirements. With regards to the financial disclosure, the funder participated in designing the study, collecting data, and analyzing results, while the researchers’ made decisions regarding publication and manuscript preparation. We have added the amended Funder statement to the cover later as well as included it in the revised manuscript (page 16-17, “Funding Disclosure” section).

3. *We note that your Data Availability Statement is currently as follows: All relevant data are within the manuscript and its Supporting Information files*

*Please confirm at this time whether or not your submission contains all raw data required to replicate the results of your study. Authors must share the “minimal data set” for their submission. PLOS defines the minimal data set to consist of the data required to replicate all study findings reported in the article, as well as related metadata and methods (*[*https://journals.plos.org/plosone/s/data-availability#loc-minimal-data-set-definition*](https://journals.plos.org/plosone/s/data-availability#loc-minimal-data-set-definition)*).*

Response: Your comprehensive review and valuable feedback on our manuscript are sincerely appreciated. We have submitted all the raw data as ‘Supporting Information’, which includes an analysis database (AnaData) in XPT format, in compliance with PLOS ONE’s requirements for a ‘minimal data set’. To facilitate accessibility and accommodate format compatibility and conversion of the XPT file, we have also provided an Excel file containing the same data. Both files will be uploaded as Supporting Information during the submission process. Please let us know if any further clarification or additional information is needed.

*4. PLOS requires an ORCID iD for the corresponding author in Editorial Manager on papers submitted after December 6th, 2016. Please ensure that you have an ORCID iD and that it is validated in Editorial Manager. To do this, go to ‘Update my Information’ (in the upper left-hand corner of the main menu), and click on the Fetch/Validate link next to the ORCID field. This will take you to the ORCID site and allow you to create a new iD or authenticate a pre-existing iD in Editorial Manager.*

Response: Thank you for your guidance. It has been validated in the Editorial Manager system.

*5. We note you have included a table to which you do not refer in the text of your manuscript. Please ensure that you refer to Table 4 in your text; if accepted, production will need this reference to link the reader to the Table.*

Response: Your thorough review of our manuscript is greatly appreciated. Table 4 presents the secondary endpoints of our study. We have amended the manuscript accordingly and cited it at the corresponding location of the results section (page 11, first paragraph of the “Secondary endpoints” section).

**Added notes**

Secondary endpoints are presented in Table 4.

**Reviewer #1: Interesting paper. Some issues should be addressed**

***Comment 1: 164 patients in 20 centers means a small number of patients for center. How were centers selected?***

**Response:** Thank you for your thorough review of our manuscript. This study initially enrolled tertiary hospitals from multiple provinces in China, selected based on their sufficient annual caseloads of tricuspid regurgitation surgeries. Based on the conducted statistical calculations, a total sample size of 164 patients was determined, with 82 patients allocated to each group, accounting for an anticipated 10% dropout rate. A strategy of site competition for enrollment was implemented during the study. Due to varying timelines for ethics approval across centers, some sites with longer review processes had limited time for patient recruitment. Nevertheless, the number of enrolled cases met the statistical requirements for non-inferiority testing (one-sided α = 0.025, β = 0.2). Additionally, all participating centers received standardized training in accordance with Good Clinical Practice (GCP) guidelines and surgical protocols to ensure consistency and quality in study conduct.

***Comment 2: definition of success rate should be added***

Response: We thank the reviewer for the valuable comment. The definition of success rate is mentioned in the ‘Endpoints’ section of the Methods. Specifically, valve repair success at six months post-surgery was defined as the absence of severe tricuspid regurgitation on echocardiography and an increase in annular diameter of no more than 15% compared to the immediate postoperative measurement. This definition reflects established criteria used in previous studies. We have slightly amended the wording of the manuscript to emphasized the definition and make it clearer (page 7, first paragraph of the “Endpoints” section).

**Revised sentence**

The primary endpoint was the success rate of valve repair six months post-surgery. The success rate was defined by two criteria: non-occurrence of severe TR as verified by cardiac ultrasound, and postoperative increase in annular diameter not exceeding 15% [1, 2]. This definition reflects established criteria used in previous studies [1, 2].

***Comment 3: Due to small number of patients, normal distribution should be checked for
clustering with ML has recently been proposes for a risk stratification for MR. Please quote and comment on PMID 40395430***

Response: The insightful comments and guidance are greatly appreciated. Quantitative data were assessed for normality using the Kolmogorov-Smirnov test, and this has been added to the amended manuscript and clarified in the Statistical Analysis section (page 8, second paragraph of the “Statistical analysis” section).

We thank the reviewer for highlighting the relevance of the study (PMID: 40395430). We agree that this work offers valuable insights into how retrospective analysis can identify factors associated with clinically meaningful outcomes following valve interventions. We have cited it and briefly mentioned its relevance to our study in the revised manuscript (page 15-16, fifth paragraph of the “Discussion” section).

**Added notes**

Furthermore, post hoc analyses of clinical trial populations can be valuable in identifying predictors of long-term outcomes. A recent study by Wedin et al. used machine learning techniques to stratify risk in patients with functional mitral regurgitation undergoing TEER, with primary outcomes of cardiovascular death or hospitalization for heart failure (HF) at one year [3]. The retrospective analysis of data from this study population is expected, aiming to explore factors influencing other clinically meaningful outcomes after surgery, and similarly, retrospective analysis of data from this study may help identify risk factors associated with TR recurrence, functional deterioration, or the need for reintervention, thereby informing patient selection and long-term management strategies in future research.

**Reviewer #2:**

**The RCT is designed to evaluate the safety and efficacy of PM-AR-T. Following are comments to be addressed.**

***Comment 1: The manuscript designed with 1:1 randomization how ever there is no information How patient were randomized between investigational device and control.***

**Response:** We thank the reviewer for their guidance and for the continued insightful input. Details regarding the randomization process are mentioned in the *Methods* section, in the procedures section. Specifically, the study implemented a dynamic randomization technique, stratifying participants by treatment center and severity of regurgitation (mild, moderate, or severe). Randomization sequences were generated by the Clinical Trial Central Randomization System (DAS for IWRS). To clarify, the process included the researchers accessed this web-based system and input the required patient information (such as identifiers and stratification factors). The system then returned the treatment assignment in real time, based on a pre-specified randomization algorithm incorporating stratification and dynamic allocation to ensure balance between groups. We have expanded this explanation in the revised manuscript to enhance clarity (page 6, first paragraph of the “Procedure” section).

**Added notes**

The randomization process involved accessing the web-based system and inputting the required patient information. The system then returned the treatment assignment in real time, based on a pre-specified randomization algorithm incorporating stratification and dynamic allocation to ensure balance between groups.

***Comment 2:*** ***Introduction section needs more strengthening e.g. side-by-side technical specification and visual comparison of design and pointing features of investigational device and control.***

**Response:** We sincerely thank the reviewer for the constructive feedback. In light of space constraints within the Introduction section, we have added detailed Supplementary Materials to enhance the reader’s understanding of the design and functional distinctions between the two devices. These materials include:

- **Textual Description of PM-AR-T (Section 1)**: This section outlines the structural and functional features of PM-AR-T, including the device characteristics, as well as its structural composition, operating principle, and mechanism of action.
- **Supplementary Figure 1:** Schematic Diagram of PM-AR-T – This figure illustrates the overall Structural components of the PM-AR-T ring, including a device assembly schematic as well as a composite schematic of the annuloplasty ring with holder, a holder handle assembly schematic, and the annuloplasty ring.
- **Supplementary Table 1:** Technical Specification Comparison Table – This table presents a comparison of PM-AR-T and the Edwards MC3.
- **Photographs of Devices/Supplementary Figure 2** – Providing a direct visual comparison of the devices.

We acknowledge the reviewer’s valid point regarding the importance of side-by-side technical specification comparisons for contextualizing this study, and have accordingly provided Supplementary Table 1. However, it should be noted that annuloplasty ring sizing is determined through brand-specific gauge measurements prior to implantation, with each gauge calibrated to its corresponding ring model. Consequently, surgeons may select different ring brands and sizes for the same patient case based on proprietary sizing systems. Such cross-brand specification comparisons offer limited insight into device distinctions. The fundamental differences between these annuloplasty rings are more significantly reflected in their structural design paradigms. Native valve annuli exhibit three-dimensional, multi-directional motion. To accommodate this dynamic coordination, the PM-AR-T incorporates a tubular lattice pattern filled with flexible wires (Supplementary Figure 2). Compared to the solid rectangular cross-section of the material-matched Edwards MC3 ring, this design enhances freedom of motion at the septal portion, better conforming to native annular dynamics. Furthermore, the PM-AR-T features graded stiffness transition along the anterior-posterior (AP) axis, with maximal rigidity at the non-patterned segment within the AP region extending toward the opposite side (Supplementary Figure 2E). This design enables targeted correction of pathological dilation occurring along the AP direction.

To improve clarity in the manuscript, we have also added a summary in the Introduction section that outlines the differences between the two devices (page 4, third paragraph of the “Introduction” section).

**Added notes**

The PM-AR-T incorporates a tubular lattice pattern filled with flexible wires. Compared to the solid rectangular cross-section of the material-matched Edwards MC3 ring, this design enhances freedom of motion at the septal portion, better conforming to native annular dynamics. Furthermore, the PM-AR-T features graded stiffness transition along the anterior-posterior (AP) axis, with maximal rigidity at the non-patterned segment within the AP region extending toward the opposite side. This design enables targeted correction of pathological dilation occurring along the AP direction.

***Comment 3: Summary of prior results of investigational device.***

**Response:** We sincerely thank the reviewer for the constructive feedback. Regarding prior studies, we would like to clarify that there are currently no published clinical articles on PM-AR-T. Prior to conducting this non-inferiority, randomized controlled trial, the device had already undergone testing at the Jinan Quality Supervision and Inspection Center for Medical Devices under National Medical Products Administration in China and received a certificate of compliance. In addition, animal experiments were completed at the Animal Experimental Center of Fuwai Hospital, Chinese Academy of Medical Sciences. The study achieved its intended objectives, and the results met the requirements for biological reproducibility and conformed to the relevant provisions of the Chinese national standard “Cardiovascular Implants—Artificial Heart Valves” (GB12279-2008). However, the detailed findings from these animal studies have not yet been published. Therefore, we have included a brief description of the animal study results in this response letter.

The Animal Experiment Center of Fuwai Hospital, Chinese Academy of Medical Sciences—which is designated as the Beijing Key Laboratory for Preclinical Studies on Cardiovascular Implantable Materials—conducted a comparative evaluation of the PM-AR-T and Edwards MC3 annuloplasty rings using small-tailed Han sheep. Briefly, a total of 29 healthy sheep underwent annuloplasty ring implantation: 17 in the PM-AR-T group (Group A) and 12 in the Edwards MC3 group (Group B). One additional animal was euthanized intraoperatively due to extensive pericardial adhesions and did not receive a ring implant. Of the remaining animals, 21 survived to the predefined study endpoints: 5 from Group A and 4 from Group B were followed up to 90 days postoperatively (±7 days), while 7 from Group A and 5 from Group B were followed up to 180 days postoperatively (±7 days). Eight animals (5 from Group A and 3 from Group B) did not survive to their respective endpoints. The surgical mortality rates were comparable between the two groups—29.0% in Group A and 25.0% in Group B—and were attributed to pre-existing conditions, surgical trauma, or inadequate postoperative care. These deaths were deemed unrelated to the implanted devices and were excluded from endpoint analyses.

The 21 surviving sheep completed weight follow-ups according to the study protocol. From surgery day to study completion, the average body weight changes trended similarly in both groups, with an initial decrease in the first week postoperatively, followed by gradual recovery and weight gain (P_time_<0.001, P_group_=0.408). Complete blood counts, biochemical tests, and coagulation function assessments were conducted preoperatively and at various follow-up time points (postoperative days 7, 30, 90, and 180) for all 21 sheep. Echocardiography and FHb testing were performed preoperatively and at each follow-up time point (immediately postoperative, postoperative days 7, 30, 90, and 180). Histopathological evaluations were carried out at 90 and 180 days postoperatively, with gross pathological assessment after euthanasia at 180 days. The echocardiographic results (Table 1) showed no tricuspid regurgitation or thrombus formation in either group. Histopathological findings (Table 2) indicated that the endothelialization degree, inflammatory response, and biocompatibility of the Puhui cardiac annuloplasty ring (tricuspid annuloplasty ring) were comparable to those of Edwards MC3 rings. Gross and histopathological examinations of hearts, lungs, kidneys, liver, spleen, and brain from all animals revealed no significant differences in postoperative complications or spontaneous disease incidence between groups, with no complications related to the annuloplasty rings. Pathological autopsies confirmed that both types of rings were properly placed in the tricuspid valve position without damage to the tricuspid leaflets or coronary arteries, and no intracardiac thrombi were observed. Due to space limitations, additional test results are not presented here but were consistent between the two groups and further support the comparable safety and performance of the two devices.

Table 1. Echocardiographic Findings in Animals

| Group | Variables | Preoperative | Immediate postoperative | 7±2 d | 30±5 d | 90±7 d | 180±7 d |
| --- | --- | --- | --- | --- | --- | --- | --- |
| A | TVPG (mmHg) | 2.80 ±1.12 | 5.19 ±3.09 | 5.57 ±6.06 | 4.16 ±2.35 | 4.01 ±1.64 | 2.90 ±1.21 |
|  | PVPG (mmHg) | 2.62 ±1.19 | 3.48 ±1.47 | 2.70 ±0.67 | 2.64 ±0.92 | 2.76 ±1.25 | 5.14 ±1.57 |
|  | EF (％) | 62.7 ±4.9 | 62.7 ±8.8 | 57.7 ±6.8 | 59.6 ±8.0 | 62.6 ±7.0 | 56.0 ±8.8 |
|  | Vertical dimension of the right atrium (mm) | 29 ±6 | 30 ±5 | 36 ±4 | 27±15 | 33 ±3 | 34 ±5 |
|  | Horizontal dimension of the right atrium (mm) | 26 ±2 | 29 ±3 | 31 ±3 | 30±12 | 31 ±3 | 32 ±6 |
|  | PAD (mm) | 18 ±2 | 21 ±2 | 20 ±2 | 20 ±2 | 21 ±2 | 22 ±2 |
|  | LVEDD (mm) | 40 ±5 | 35 ±5 | 41 ±7 | 44 ±3 | 46 ±5 | 46 ±6 |
|  | LVESD (mm) | 24 ±5 | 23 ±4 | 28 ±7 | 28 ±3 | 29 ±3 | 30 ±7 |
| B | TVPG (mmHg) | 3.34 ±0.88 | 4.48 ±1.96 | 4.80 ±2.72 | 6.43 ±3.01 | 4.64 ±1.86 | 3.12 ±0.93 |
|  | PVPG (mmHg) | 2.44 ±0.40 | 3.95 ±1.55 | 3.46 ±1.62 | 3.47 ±1.35 | 3.24 ±1.05 | 5.67 ±3.81 |
|  | EF (％) | 66.6 ±6.3 | 63.9 ±9.1 | 57.4 ±5.0 | 63.0 ±8.0 | 59.4 ±4.4 | 61.8 ±8.2 |
|  | Vertical dimension of the right atrium (mm) | 30 ±4 | 30 ±4 | 34 ±6 | 33 ±5 | 32 ±5 | 35 ±3 |
|  | Horizontal dimension of the right atrium (mm) | 28 ±4 | 27 ±2 | 31 ±4 | 29 ±6 | 28 ±5 | 33 ±3 |
|  | PAD (mm) | 19 ±2 | 18 ±1 | 20 ±1 | 20 ±1 | 22 ±1 | 22 ±1 |
|  | LVEDD (mm) | 36 ±6 | 37 ±4 | 40 ±4 | 40 ±6 | 40 ±7 | 44 ±6 |
|  | LVESD (mm) | 20 ±6 | 23 ±3 | 27 ±4 | 26 ±4 | 23 ±6 | 26 ±4 |

Table 2. Histopathological Evaluation Results

| Number | Animal ID | Group | Thrombus formation | Endothelialization rate | Inflammation score | Fibrous tissue growth grade | Biocompatibility |
| --- | --- | --- | --- | --- | --- | --- | --- |
| 1 | S2018-121 | A（180±7 d） | No | 100% | 4 | 2 | Good |
| 2 | S2019-015 | A（180±7 d） | No | 10% | 4 | 2 | Acceptable |
| 3 | S2019-018 | A（180±7 d） | No | 100% | 4 | 2 | Good |
| 4 | S2019-021 | A（180±7 d） | No | 100% | 4 | 2 | Good |
| 5 | S2019-023 | A（180±7 d） | No | 80% | 5 | 2 | Good |
| 6 | S2019-027 | A（180±7 d） | No | 100% | 4 | 2 | Good |
| 7 | S2019-114 | A（180±7 d） | No | 30% | 8 | 2 | Acceptable |
| 8 | S2019-108 | A（180±7 d） | No | 30% | 8 | 2 | Acceptable |
| 9 | S2019-109 | B（180±7 d） | No | 90% | 3 | 2 | Good |
| 10 | S2019-110 | B（180±7 d） | No | 90% | 4 | 1 | Good |
| 11 | S2019-115 | B（180±7 d） | No | 100% | 8 | 2 | Good |
| 12 | S2019-116 | B（180±7 d） | No | 100% | 4 | 2 | Good |
| 13 | S2019-120 | A（90±7 d） | No | 90% | 3 | 2 | Good |
| 14 | S2019-122 | A（90±7 d） | No | 85% | 3 | 2 | Good |
| 15 | S2019-154 | A（90±7 d） | No | 95% | 4 | 2 | Good |
| 16 | S2019-155 | A（90±7 d） | No | 95% | 4 | 2 | Good |
| 17 | S2019-156 | A（90±7 d） | No | 100% | 3 | 2 | Good |
| 18 | S2019-158 | B（90±7 d） | No | 100% | 3 | 2 | Good |
| 19 | S2019-161 | A（90±7 d） | No | 100% | 5 | 2 | Good |
| 20 | S2019-162 | A（90±7 d） | No | 100% | 3 | 2 | Good |
| 21 | S2019-163 | A（90±7 d） | No | 90% | 4 | 2 | Good |

To improve clarity in the manuscript, we have also added a summary in the Introduction section that outlines the detailed results of animal study (page 4, third paragraph of the “Introduction” section).

**Revised sentence**

Preliminary studies in animal models (unpublished data), specifically small-tailed Han sheep, demonstrated comparable outcomes between PM-AR-T (n=17) and Edwards MC3 rings (n=12). Surgical mortality (PM-AR-T: 29.0%, Edwards MC3: 25.0%) was unrelated to devices. Among 21 survivors reaching endpoints (90±7d: PM-AR-T, n=5, Edwards MC3, n=4; 180±7d: PM-AR-T, n=7, Edwards MC3, n=5), echocardiography revealed no tricuspid regurgitation or thrombus in either group. Histopathology [4] indicated equivalent endothelialization, inflammatory responses, and biocompatibility. Gross pathology confirmed correct ring positioning without leaflet/coronary damage or device-related complications.

***Comment 4: Reference 15 coated animal study of test device, however detailed results in manuscript body would be helpful for readers. Moreover, providing DOI in reference section would help to read detailed results about test device.***

Response: We sincerely appreciate the reviewer’s thorough evaluation of our manuscript. Reference 15 is a Chinese-language article, and therefore, although we have provided its DOI, it may not be accessible through databases such as PubMed, and an English translation may not be available—we apologize for this inconvenience, and we have therefore included a description of it in this response letter. Reference 15 is a methodology paper that describes the pathological evaluation methods used in preclinical animal studies of valve annuloplasty rings. Briefly, two Chinese-made annuloplasty rings and their control products were surgically implanted into the mitral or tricuspid valve positions of 17 adult small-tailed Han sheep (experimental group) and 5 sheep (control group). The animals were euthanized 20 weeks post-surgery, and assessments were conducted through gross observation, histopathological analysis, and scanning electron microscopy. These assessments focused on surgical-related injuries, ring integrity, inflammatory responses, endothelialization, fibrous sheath formation, excessive fibrous tissue growth, and thrombus formation. Additionally, organs such as the heart, liver, spleen, lung, kidney, and brain were evaluated for infarction and infection. For animals that died before the study endpoint, cause-of-death analyses were performed to determine any relationship between death and the annuloplasty rings, thereby evaluating the device’s safety. Histopathology and related assessments for preliminary studies in animal models at Fuwai Hospital’s Animal Experiment Center were conducted according to this methodological reference. Detailed results of the PM-AR-T animal study are presented in the third paragraph of the Introduction section.

***Comment 5: Any COVID related assessment on outcome would be helpful.***

Response: We appreciate the reviewer’s comment and agree that COVID-related assessments on outcome would be helpful. It is important to note that the COVID-19 outbreak took place during the study period. However, recruitment of new patients was halted, and the patients who were already enrolled chose to proceed with elective surgery when the outbreak affected all centers. As a result, no COVID-related outcomes were assessed.

***Comment 6: A brief information of data evaluation, data safety monitoring committee and steering committee would be useful for readers.***

Response: We sincerely appreciate the valuable suggestion regarding oversight committees. This study did not convene a formal Data Safety Monitoring Board (DSMB), while we implemented a Clinical Endpoints Committee (CEC) to ensure rigorous endpoint adjudication. The CEC comprised independent clinical experts unaffiliated with the trial, who conducted review of all investigator-reported endpoints using predefined criteria. We have added a summary in the manuscript (page 7, third paragraph of the “Endpoints” section).

**Added notes**

An independent Clinical Endpoints Committee (CEC) reviewed all investigator-reported endpoints.

***Comment 7: Raw data sheet for statistical calculation should be available as supplementary data sheet.***

Response: Thank you for your continued valuable guidance. We have submitted all the raw data as ‘Supporting Information’, which includes an analysis database (AnaData) in XPT format, in compliance with PLOS ONE’s requirements for a ‘minimal data set’. To facilitate accessibility and accommodate format compatibility and conversion of the XPT file, we have also provided an Excel file containing the same data. Both files will be uploaded as Supporting Information during the submission process.

**References**

1.	Meng H, Wang H, Pan S, Feng K, Li J, Wang Y. The Insight into Secondary Tricuspid Regurgitation by Two and Three—dimensional Echocardiography. Chin J Ultrasound Med. 2015;31(04):326-8.

2.	Zheng S, Jiao Y, Meng X. The impact of three-dimension ring on size of mitral annulus. Chin J Cardiovasc Dis. 2018;37(06):547-50. doi: 10.3969/j.issn.1007-5062.2018.06.013

3.	D'Ascenzo F, Angelini F, Pancotti C, Bocchino PP, Giannini C, Finizio F, et al. Machine-learning phenotyping of patients with functional mitral regurgitation undergoing transcatheter edge-to-edge repair: the MITRA-AI study. European heart journal Digital health. 2025;6(3):340-9. doi: 10.1093/ehjdh/ztaf006

4.	Duan X, Wang H, Tang Y, Yang B, Tang Y, Wang Q, et al. Pathological evaluation in preclinical safety study of two new annuloplasty rings at the mitral or tricuspid valve positions in sheep. Chin J Exp Surg. 2018;35(7):1234-7. doi: 10.3760/cma.j.issn.1001-9030.2018.07.012
